# Serial Recall Predicts Vocoded Sentence Recognition Across Spectral Resolutions

**DOI:** 10.1044/2020_JSLHR-19-00319

**Published:** 2020-03-26

**Authors:** Adam K. Bosen, Michael F. Barry

**Affiliations:** a Boys Town National Research Hospital, Omaha, NE

## Abstract

**Purpose:**

The goal of this study was to determine how various aspects of cognition predict speech recognition ability across different levels of speech vocoding within a single group of listeners.

**Method:**

We tested the ability of young adults (*N* = 32) with normal hearing to recognize Perceptually Robust English Sentence Test Open-set (PRESTO) sentences that were degraded with a vocoder to produce different levels of spectral resolution (16, eight, and four carrier channels). Participants also completed tests of cognition (fluid intelligence, short-term memory, and attention), which were used as predictors of sentence recognition. Sentence recognition was compared across vocoder conditions, predictors were correlated with individual differences in sentence recognition, and the relationships between predictors were characterized.

**Results:**

PRESTO sentence recognition performance declined with a decreasing number of vocoder channels, with no evident floor or ceiling performance in any condition. Individual ability to recognize PRESTO sentences was consistent relative to the group across vocoder conditions. Short-term memory, as measured with serial recall, was a moderate predictor of sentence recognition (ρ = 0.65). Serial recall performance was constant across vocoder conditions when measured with a digit span task. Fluid intelligence was marginally correlated with serial recall, but not sentence recognition. Attentional measures had no discernible relationship to sentence recognition and a marginal relationship with serial recall.

**Conclusions:**

Verbal serial recall is a substantial predictor of vocoded sentence recognition, and this predictive relationship is independent of spectral resolution. In populations that show variable speech recognition outcomes, such as listeners with cochlear implants, it should be possible to account for the independent effects of spectral resolution and verbal serial recall in their speech recognition ability.

**Supplemental Material:**

https://doi.org/10.23641/asha.12021051

When speech is easy to understand, listeners can rapidly and automatically identify speech cues, but in difficult listening conditions, additional cognitive processes must be engaged to support speech recognition (Rönnberg et al., 2013; Wingfield et al., 2015). This relationship is most evident in listeners with hearing loss, who often struggle with speech recognition even if they use hearing aids or cochlear implants. Listeners with hearing loss often differ from listeners with typical hearing in their relationships between speech recognition and various measures of cognition, such as fluid intelligence, memory, and attention (Kaandorp et al., 2017; Kronenberger et al., 2014; Moberly, Harris, et al., 2017; Moberly et al., 2016; Moberly, Houston, et al., 2017; O'Neill et al., 2019; G. N. Smith et al., 2019). Currently, it is unclear how to interpret these differences across groups of listeners. Changes in the relationship between cognition and speech recognition across listeners with normal hearing and listeners with hearing loss could be due to fundamental changes in how listeners with hearing loss use their cognitive abilities to process speech. Alternatively, these relationships could be moderated by differences in age or hearing status across tested groups, which would confound comparison across groups. Additionally, both speech recognition and cognition are composed of several distinct but related processes. Characterizing the relationship between speech recognition and cognition requires understanding what latent constructs are engaged by the tasks used in an experiment. To address these issues, we tested vocoded sentence recognition across three levels of spectral resolution within the same group of young adults and compared individual differences in sentence recognition to a set of tasks designed to measure distinct aspects of cognition. Below, we demonstrate that testing speech recognition in the same individuals across different task difficulties enabled us to determine that relationships between speech recognition and cognition did not change with spectral resolution. Splitting cognitive tasks to cover distinct processes demonstrated that serial recall is the primary ability that predicts vocoded sentence recognition in these listeners.

## Differences in Cognition Across Speech Recognition Tasks

For speech recognition, a common, clinically relevant task is to identify words or sentences in some form of competing noise. Recognizing speech in noise requires segregating auditory streams from one another, attending the relevant stream, and identifying speech cues in the partially masked relevant stream (for a review, see Shinn-Cunningham & Best, 2008). Speech recognition performance is not correlated across speech-in-noise maskers and speech-in-speech maskers or between native and nonnative speech recognition (McLaughlin et al., 2018), which indicates that the skills required for listening in different adverse conditions depend on the adverse condition (see also Mattys et al., 2012, for a review). The extent to which these cognitive factors are relied upon also depends on the complexity of the speech task. Tasks with little linguistic processing, such as discriminating two phonemes from one another, show little relationship with aspects of cognition, whereas tasks that include more linguistic elements, such as word or sentence recognition, are correlated with measures of memory and attention (Heinrich et al., 2015, 2016). To simplify the number of factors at play in this study, we used vocoded sentences to control speech recognition difficulty. This approach used a single auditory stream and therefore did not necessitate stream segregation and the associated attentional mechanisms, while still allowing for semantic and lexical processing of meaningful speech. The removal of this stream segregation requirement likely changes the cognitive factors at play in speech recognition, so it is possible that the cognitive factors that support speech recognition will differ between this study and previous studies of speech in noise. To assess this possibility, we used a battery of tasks designed to test fluid intelligence, short-term memory, and attention.

## Fluid Intelligence

The rise of cognitive hearing science (Arlinger et al., 2009) has emphasized the role of cognition in speech recognition, but previous studies have not always clearly distinguished between distinct elements of cognition and the processes required to successfully perform a speech recognition task. Some studies have found that a single overall “cognition” factor predicts some of the variance in speech recognition in noise (Humes et al., 2013; Rönnberg et al., 2016; van Rooij & Plomp, 1990). A recent meta-analysis by Dryden et al. (2017) showed that most groupings of cognitive tasks by subdomains, such as inhibitory control and working memory, produced similar correlations between different subdomains and sentence in noise recognition. A parsimonious explanation for these similar correlations is a first-order construct, fluid intelligence, which captures general variability in cognitive ability across subdomains. Here, we used the Matrix Reasoning and Block Design subscales of the Wechsler Abbreviated Scale of Intelligence–Second Edition (WASI-II; Wechsler, 2011). These subscales both load similarly on the same latent construct, which has been conceptualized as fluid intelligence or, more specifically, perceptual organization/reasoning (Gignac, 2014; Humes et al., 2007; Tulsky & Price, 2003). As an alternative, cognition can be conceptualized as having multiple core facets (e.g., Friedman et al., 2008; Miyake & Friedman, 2012; Miyake et al., 2000). Speech recognition may associate to different degrees with each of these facets, so we additionally consider tests of memory and attention.

## Short-Term Memory

Reading span (Daneman & Carpenter, 1980) is common test of verbal working memory, which has successfully predicted various speech recognition in noise outcomes (for reviews, see Akeroyd 2008; Dryden et al. 2017). Reading span is a form of complex span task, in which participants must alternate between storing successive items in memory and processing unrelated information (Conway et al., 2005). Reading span is a complex task that engages several distinct aspects of cognition (Diamond, 2013; Engle & Kane, 2004). When considering the structure of memory, a common model is the combination of a primary focus of attention that can only hold a few items at once, a secondary set of activated long-term memory (Cowan, 2001; Oberauer, 2002; Unsworth & Engle, 2007b), and an attentional control component that coordinates action between the two stores (Shipstead et al., 2014). In complex span tasks, items to be stored are briefly moved into the focus of attention, displaced to secondary memory when processing subsequent information, and retrieved for rehearsal during the trial and for recall at the end of the trial. This process heavily taps into an individual's ability to use secondary memory (Unsworth & Engle, 2007a), as well as their ability to build, maintain, and update a sequence of information (Wilhelm et al., 2013). Working memory capacity is closely associated with fluid intelligence (Colom et al., 2015; Engle & Kane, 2004; Kane et al., 2005; Shipstead et al., 2016) because working memory relies heavily on cognitive control/executive attention to manipulate information held in memory (Conway & Kovacs, 2013; Engle & Kane, 2004; McCabe et al., 2010). Fluid intelligence has been demonstrated to be correlated with and distinct from working memory (Engle & Kane, 2004; Gignac, 2014; Shelton et al., 2010; Wilhelm et al., 2013). Therefore, it is possible that previously observed correlations between working memory and speech recognition may reflect their mutual association with fluid intelligence.

To test memory, we opted to use auditory serial recall tasks because they are conceptually similar to sentence recognition tasks. In typical speech recognition and serial recall tasks, speech is presented and held in memory until response, without distracting tasks competing for attention. Additionally, in both tasks, the instructions to the participant are to simply listen to and repeat what they heard. Serial recall and complex span tasks largely measure the same underlying constructs but differ in the extent to which they engage primary memory and secondary memory (Unsworth & Engle, 2007a). However, the reliability of serial recall is strongly determined by task design (Unsworth & Engle, 2006, 2007a) and analysis (Woods et al., 2011). When recalling long lists of items, chunking supports serial recall, and the ability to chunk ongoing or long sequences of information reflects the activation of learned associations between items held in long-term memory, rather than simple passive storage (Cowan, 2001). As a result, only the variance in performance across long lists (e.g., lists of six digits or more in forward digit span) in serial recall tasks show the same associations with fluid intelligence as complex span tasks (Unsworth & Engle, 2006, 2007a). This finding indicates that adaptive serial recall tasks, which start at shorter list lengths and get longer until a criterion number of errors is made, can miss essential variance in performance at long list lengths. In addition, using the longest list length correctly recalled in adaptive serial recall as a metric of performance has low test–retest reliability (Woods et al., 2011). To address these limitations, our serial recall tasks used a fixed number of trials across a range of list lengths and scored performance based on the proportion of items correctly recalled across all list lengths.

One practical consideration in using auditory serial recall to test individuals in adverse listening conditions is to distinguish between errors that arise from speech recognition difficulties and errors that arise from the limitations of memory. In our previous work (Bosen & Luckasen, 2019), we demonstrated that listener ability to remember and repeat lists of digits was unimpaired by limiting spectral resolution with an eight-channel vocoder, whereas performance for lists of words was impaired by vocoding. We found that two principal components accounted for most of the variance in serial recall performance across stimuli and listening conditions. The largest component, which explained about 67% the variance, loaded equally across digits, words, and nonwords in both clear and vocoded listening conditions. This equal loading indicates that this component reflects individual ability to perform serial recall in general. The second component, which explained about 17% of the variance, loaded specifically on serial recall of vocoded words and nonwords. Therefore, this component appears to reflect the distinct impairment imposed by alternating between identifying vocoded items as they were presented and storing the preceding list of items. For digits and words presented clearly, this alternation is trivial, because items can be readily identified. However, for vocoded words, the input becomes ambiguous, and explicit processing (Rönnberg et al., 2013) is necessary to identify items. Using both digits and unrelated words for serial recall across listening conditions will enable us to dissociate these two previously identified components. Our hypothesis was that the impairment imposed by identifying degraded items (the second component) would be the best predictor of vocoded sentence recognition, although our results indicate this was not the case.

## Attention

To test attention, we included tasks to measure attentional switching, inhibition, and sustained attention. Overall, maintaining sequences in memory is an attentional demanding process (Chen & Cowan, 2009), and attentional control also has distinct relationships with working memory and fluid intelligence (Unsworth et al., 2009), so it is possible that attention will associate with speech recognition and the other cognitive tests used here. With respect to speech recognition, short-term memory and speech recognition mutually influence each other in a manner that may be influenced by attentional switching. When items are difficult to identify, listeners must allocate cognitive resources to identification, which reduces their ability to use those resources to store and retrieve items they have already heard (Luce et al., 1983; Pichora-Fuller et al., 1995). Conversely, maintaining a list of items in memory impairs recognition of words in vocoded listening conditions (Hunter & Pisoni, 2018). Based on mutual interference between memory maintenance and word identification, attentional switching may be required to facilitate successfully sharing cognitive resources across both tasks (Barrouillet et al., 2004, 2007). Here, attentional switching was assessed with a color–shape categorization task (Miyake et al., 2004).

In listeners with cochlear implants, reaction times in an incongruent color Stroop task has been shown to correlate with speech recognition (Moberly, Houston, et al., 2017), which could reflect the role of inhibition in understanding speech with a cochlear implant. To test the possibility that this result will replicate in our listeners, we used the same Stroop task. Previous research has indicated that the size of the long tail of the distribution of an individual's reaction times is strongly associated with working memory (Schmiedek et al., 2007). This long tail characterizes trials in which the response was slower than typical, and as such, the size of long tail can be interpreted as the rate at which individuals have brief attentional lapses during a task. For the color–shape categorization and Stroop tasks, we included enough trials to provide an unbiased estimate of the size of the long tail.

Sustained attention was assessed with the Test of Variables of Attention (TOVA; Greenberg, 2011). Although performance on the TOVA is not strongly correlated with serial recall performance (Bosen & Luckasen, 2019; Kronenberger et al., 2014), performance on the TOVA differs between listeners with cochlear implants and listeners with typical hearing (Kronenberger et al., 2013). Therefore, the TOVA is included not because we expect it to strongly correlate with vocoded speech recognition or other tests of cognition but rather to rule out sustained attention as an explanation for individual differences in speech recognition.

## Challenges in Comparing Across Groups

Differences in age between observed groups could produce apparent differences in the relationship between hearing loss and cognition, regardless of hearing status. Listeners with hearing loss tend to be older, and older adults with low memory ability show greater impairment in sentence recognition than younger adults with matched memory ability (Gordon-Salant & Cole, 2016). As a result, the effect of memory on sentence recognition would be larger in older adults than in young adults. Other difficulties, such as an increased impairment from proactive interference in memory tasks (Emery et al., 2008), may also occur with age and moderate the relationship between tests of speech and cognition. The possibility of additional moderators arising with age makes it difficult to compare explanatory factors across groups of different ages.

Aging also produces a complex, interlinked decline in perception and cognition (Loughrey et al., 2018; Roberts & Allen, 2016), with a variable trajectory for every individual. Sensory impairments can reduce performance on tasks that are primarily designed to be tests of cognition (Baldwin & Ash, 2011; Dupuis et al., 2015; Guerreiro & Van Gerven, 2017; McCoy et al., 2005). In listeners with hearing loss, this effect of sensory impairment can make it difficult to distinguish cognitive and sensory impairments from one another. The difficulty in distinguishing these impairments from one another is problematic because both contribute to speech perception (Humes et al., 2013; Lunner, 2003; van Rooij & Plomp, 1990), so it can be difficult to correctly attribute speech perception difficulties to cognitive or sensory origins. Additionally, the range of individual variability in sensory impairment differs across listeners with hearing loss and listeners with normal hearing. As a result, listeners with hearing loss often show greater variability in their speech recognition outcomes than listeners with normal hearing (see G. N. Smith et al., 2019, as a recent example). A group with a wider range of performance will have apparently stronger correlations than a group with a narrower range. These effects indicate that hearing loss influences many facets of experimental outcomes, which could produce apparent differences in correlation strengths across groups with and without hearing loss. To address this possibility, here we focus on speech recognition across conditions of varying difficulty within the same group of listeners.

## Current Objective

The goal of this work was to determine if the relationship between components of cognition and speech recognition changes with spectral resolution within the same group of individuals. We hypothesized that relationships between components of cognition and speech recognition should only appear when spectral resolution is sufficiently degraded to necessitate reliance on cognition in the first place. This hypothesis was based on the fact that, in good quality listening conditions, identifying speech is easy, and so little explicit processing of speech cues is necessary (Rönnberg et al., 2013). To test this hypothesis, we measured speech recognition across three difficulty levels, produced by vocoding, as well as individual differences in fluid intelligence, short-term memory, and attention. Because we tested the same group of younger adults across multiple listening conditions, the pattern of correlations we observed is likely to reflect relationships to mechanisms specifically associated with speech recognition, rather than proxy measures of cross-group differences or general age-related decline.

## Method

Young adults with typical sensory and neurological function repeated sentences processed through three different vocoders of varying spectral resolution (4-, 8-, and 16-channel noise band vocoders). Each participant also completed tests of fluid intelligence (WASI-II), short-term memory (digit and word serial recall), attentional switching (color–shape task), inhibition (color Stroop task), and sustained attention (TOVA). Performance in each task was compared across individuals and spectral resolutions to determine how these aspects of cognition influence speech recognition at different spectral resolutions.

### Participants

Thirty-eight young adults (12 men, 19–29 years of age) were recruited by the Human Subjects Core at Boys Town National Research Hospital to participate in this study. Of these 38 young adults, six did not complete both experimental sessions, leaving 32 participants for the comparison between speech recognition and cognitive abilities. One participant did not complete the TOVA task due to self-reported photosensitivity. All participants were screened for typical hearing (pure-tone thresholds < 20 dB HL at octave frequencies between 0.5 and 8 kHz) and normal or corrected-to-normal vision (visual acuity of 20/20 or 20/25) and did not report any developmental, intellectual, or neurological disorders that would interfere with any of the tests used here. Participants provided written informed consent and were compensated hourly for participation. This study was approved by the Boys Town National Research Hospital Institutional Review Board and was conducted in the Lied Learning and Technology Center.

### Sentence Recognition

Participants repeated Perceptually Robust English Sentence Test Open-set (PRESTO) sentences (Gilbert et al., 2013; Tamati et al., 2013). These sentences were used because talker gender, talker dialect, syntactic structure, and semantic contents vary between sentences, which prevents strategic use of these cues to process speech. Two sentence lists were used for each spectral resolution. Pairs of lists were selected to be approximately equal in difficulty with an eight-channel vocoder (Faulkner et al., 2015). Lists 7 and 15 were used in the 16-channel vocoder condition, Lists 13 and 17 were used in the eight-channel vocoder condition, and Lists 8 and 23 were used in the four-channel vocoder condition. Each list contains a set of 18 sentences with 76 key words that are unevenly distributed across sentences, for a total of 152 key words per spectral resolution.

Sentences were presented in an echo-attenuated sound booth from a loudspeaker approximately 1 m straight ahead of participants. Presentation level was adjusted such that the long-term average spectrum of all auditory material was at 65 dB SPL. For each sentence, participants clicked a button on a personal computer to play the sentence once and then repeated back what they heard. No feedback was provided, and no time limit was placed on responses. Verbal responses were scored by an experimenter during the experiment and were recorded for later reference. Both authors scored results from 12 participants (four from each spectral resolution) for validation. Scores given by each author differed by an absolute mean of 2.3% (maximum 4.6%), indicating good consistency across authors.

Spectral resolution was manipulated by processing sentences with 4-, 8-, or 16-channel noise-band carriers. These numbers of channels were selected to span a range of sentence recognition accuracy (Friesen et al., 2001). We used vocoding to manipulate spectral resolution because it allows us to precisely control the amount of information available without introducing additional sounds (e.g., background noise). We wanted to avoid introducing additional sounds to avoid engaging stream segregation. Vocoding was performed as in the study of Bosen and Chatterjee (2016). Stimuli were passed through rectangular filters with different edge frequencies that were approximately equally spaced on the Greenwood (1990) function (filter edges of 100, 164, 245, 346, 475, 637, 842, 1099, 1425, 1835, 2352, 3005, 3828, 4866, 6175, 7826, and 10000 Hz for the 16-channel vocoder, with adjacent bands combined for lower numbers of channels). The envelope of each filter's output was extracted via the Hilbert transform and then low-pass filtered with a 300-Hz fourth-order Butterworth filter. The low-pass envelopes were multiplied with band-limited noise carriers of corresponding frequency range, and the products were summed across band to produce the vocoded signal. The PRESTO sentence recordings are all low-pass filtered, with a cutoff frequency of approximately 7.25 kHz, so the highest channel of the 16-channel vocoder effectively had no energy in it. However, this frequency range contributes minimally to speech recognition (Yoho et al., 2018), so the effective loss of this channel in the 16-channel vocoder is unlikely to affect performance.

### Fluid Intelligence

The WASI-II Block Design and Matrix Reasoning subtests were used to assess fluid intelligence (Wechsler, 2011). Both are visuospatial, in contrast to the verbal tasks used for speech and memory. In the Block Design task, individuals manipulate cubes with colored sides to produce a target pattern within a given time limit. Target patterns start simple and progressively get more complex until the participant is unable to complete three target patterns within the time limit or until they complete all trials. Accuracy and speed are converted to a single normed score. In the Matrix Reasoning task, participants are shown an incomplete pattern and must choose one of five possible segments that complete the pattern. Responses were scored as correct or incorrect and converted to a single normed score. Scores from both tasks were combined to provide a Perceptual Reasoning Index for each individual.

### Serial Recall

Participants repeated lists of digits and words in the same order that they were presented (forward span). Lists of digits ranged between two and nine items in length, and lists of single-syllable consonant–vowel–consonant words ranged between one and six items in length. Lists were presented using the same equipment and spectral resolutions as in the sentence recognition task. List presentation order was edited to avoid repeating the same length list twice in a row. Lists were presented in the same order for all participants. The first trial of each block was a list of at most six digits or four words to avoid discouraging participants with harder trials at the beginning of the block.

Each digit presented was randomly sampled from one of six different recorded utterances by a single female talker to provide some variability in production. Initially, we recorded 20 utterances of each digit. From those 20, the authors selected six that were clearly produced and had similar inflections. All recordings were bandpass filtered between 80 Hz and 20 kHz (through a fourth-order Butterworth filter both forward and in reverse, to avoid any phase distortion) and then normalized to have equal peak amplitude. Digit lists were edited to avoid transitions by ± 1, to avoid storage of digits as sequential chunks (Cowan, 2001).

Sixty consonant–vowel–consonant words were selected for inclusion in word span lists based on two criteria. First, words were selected that only contain phonemes from a restricted set that minimized the occurrence of phonemes from the same phonetic confusion clusters (DiNino et al., 2016). Allowed phonemes were the consonants /w/, /d/, /p/, /s/, /ʃ/, /m/, /f/, /v/, /tʃ/, /h/ (initial phoneme only), and /z/ (final phoneme only) and the vowels /ӕ/, /ɑ/, /e/, /ε/, /i/, /o/, and /u/. To obtain the desired number of words, we could not completely eliminate confusions (notably /s/ and /ʃ/, and /ӕ/ and /ɑ/), but we did avoid a number of likely confusions in vocoded speech (e.g., we only included /p/, but not /t/ or /k/). This restriction would make it easier to distinguish between phonetic confusions and failures of memory for planned future analyses that were beyond the scope of the current work. Second, words had lexical neighborhood density estimates provided by Storkel (2013) and frequency estimates provided by Brysbaert and New (2009), which allowed us to balance the distribution of these properties within and across word lists. Words with homophones used the highest frequency homophone when balancing word lists. These criteria yielded a total of 79 words, from which the final 60 were selected.

Word lists were created by combining these words such that no phoneme was repeated in the same word-level position within a list. Lists were designed to have similar lexical neighborhood densities and word frequencies across all lists and list lengths, because both properties affect serial recall accuracy (Quinlan et al., 2017; Roodenrys et al., 2002). Different lists were used in each spectral resolution. Across the experiment, each word was presented a total of 10 or 11 times, with either three or four presentations per spectral resolution. A complete set of words and word lists is available in Supplemental Material S1.

### Attention

Attentional switching was assessed with a color–shape categorization task, in which participants are prompted to sort items by either color or shape as fast as possible (Miyake et al., 2004). This task was conducted in the Inquisit 5 Lab software (Inquisit 5, 2016). Each trial started with a cue word of “COLOR” or “SHAPE” presented on a computer monitor. At 200 ms later, a red or green circle or triangle was presented, and subjects had to press either the “a” (for circle or green) or “l” (for triangle or red) key on a keyboard to categorize the item. The rate at which the cue either switched or stayed the same was balanced across the task. Response times are typically slower when the cue word switches between trials than when it repeats, which is interpreted as the cost of switching attentional focus between categorization rules. Our test corresponds to the “Control-Short Word Cue” condition in the study of Miyake et al. (2004, Figure 3). Prior to the main task, participants completed a series of training blocks that explained the task and allowed them to practice the task with both constant and switching cue words.

We wanted enough trials to accurately estimate the exponential Gaussian shape of individual reaction time distributions. Simulated sampling indicated that a minimum of 50 trials per condition was needed to provide an unbiased estimate of the size of the long tail of the distribution, so we conservatively used 80 trials per condition to ensure that we could accurately estimate the shape of the reaction time distribution. We tested 80 trials per training blocked cue conditions and 80 switch and 80 repeat trials each in the main task, for a total of 320 trials. A 1-min break was provided halfway through the main task. This task took between 14 and 29 min to complete (*Mdn* = 19 min).

Inhibition was assessed with a color categorization Stroop task (Stroop, 1935). On each trial, participants had to categorize an item on the screen as being one of four colors as fast as possible. Colors were red, green, blue, and black, corresponding to “d,” “f,” “j,” and “k” on the keyboard, and these color-to-key mappings were kept on screen throughout the task. Items were either the name of the color in text (congruent), the name of a different color (incongruent), or a sold rectangle (control). Responses in the incongruent condition are generally slower than in the congruent and control conditions, which is believed to reflect the cost of inhibiting task-irrelevant information. As in the attentional switching task, 80 trials were tested per condition, for a total of 240 trials. A break was provided halfway through the task. Equal proportions of each condition were tested, because the distribution of conditions can affect performance (Kane & Engle, 2003). Prior to the main task, participants completed a practice set to familiarize themselves with the task. Participants were required to reach 90% accuracy on 24 practice trials before they started the main task and repeated the practice until they reached 90%. This task typically took about 6 min to complete.

The TOVA is a go/no-go paradigm used to assess sustained attention (Leark et al., 2008). Administration of the TOVA started with a short training video that provided instructions for the task. The task consisted of flashes of white squares on a computer monitor. Within the white square, a smaller black square was presented either at the top or bottom of the white square. Participants pressed a button for trials in which the black square appeared at the top but had to avoid responding when the black square appeared at the bottom. The test lasted 21 min and measured response accuracy and reaction times. These measures were compared to normative samples to obtain percentile rankings for omission rate, commission rate, reaction time, and reaction time variability.

### Experiment Sequence

All participants completed the set of tasks in the same order across two experimental sessions. Session 1 started with informed consent, followed by a hearing and vision screening. Participants then completed the fluid intelligence tests, which were the Block Design and Matrix Reasoning subscales of the WASI in that order, followed by the attention tests, which were the TOVA, color–shape categorization, and Stroop, again in that order. Session 2 comprised digit recall, PRESTO sentence recognition, and word recall. In our previous study, there was a trend toward increasing item identification accuracy when participants started with the vocoded listening condition, but not when they started with the unprocessed listening condition (Bosen & Luckasen, 2019). To avoid confounding effects of item learning affecting the results, spectral resolutions were presented in blocks from easiest to hardest conditions (16-, eight-, and four-channel vocoders, in that order). Within each spectral resolution block, digit recall, sentence recognition, and word recall were performed in that order. Prior to every task, participants were shown an example of the task and were allowed to ask any clarifying questions prior to starting the task. To address possible time of day effects (Veneman et al., 2013) across experimental sessions, both sessions were scheduled as close to the same time of day as feasible for the participant's schedule. The median time between test sessions was 21 days, although due to scheduling difficulties, three participants had greater than 90 days between test sessions. Each session took between 2.5 and 3 hr to complete.

### Planned Analyses

Planned analysis of the data started with examining simple linear correlations of PRESTO sentence recognition accuracy across different levels of spectral resolution and between sentence recognition and predictor variables. Sentence recognition accuracy was quantified as the proportion of key words correct across both lists in each listening condition. For fluid intelligence, the WASI Perceptual Reasoning Index was used as the only predictor. For serial recall, the proportion of correctly recalled digits and phonemes in each listening condition was used as predictors in the first stage of analysis. In addition, we replicated an analysis previously reported by Bosen and Luckasen (2019), in which principal components analysis was used to reduce dimensionality of serial recall task performance across stimuli and listening conditions. In that study, serial recall performance across sets of digits, words, and nonwords under clear and vocoded listening conditions could be explained by two principal components. The first component reflected overall serial recall ability and loaded equally across experimental conditions. The second component reflected how much serial recall was impaired by vocoding for words and nonwords. We used these two components as predictors as well. For attention, we used the mean difference in reaction time across conditions in the color–shape categorization task and the Stroop task, as well as the four comparison to normative sample scores from the TOVA as predictors. Additional analyses were planned to examine differences in correlation strength across spectral resolutions, but the data described below did not indicate that these analyses were justified. Instead, post hoc analyses were conducted as described below. This study was not preregistered. All data and analysis scripts are available as Supplemental Material S1.

## Results

### Sentence Recognition

**Figure 1.**
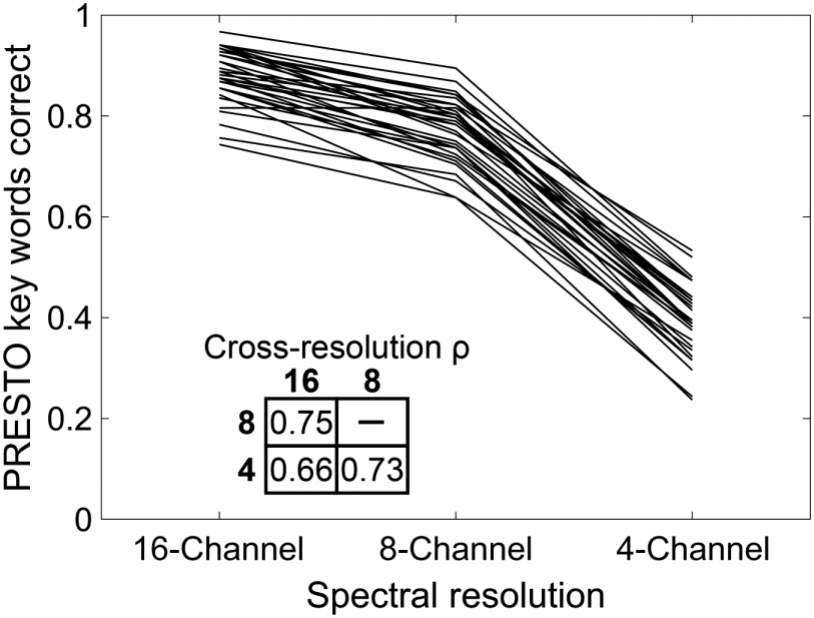
Perceptually Robust English Sentence Test Open-set (PRESTO) sentence recognition across vocoder spectral resolutions. Each line represents the proportion of key words one individual correctly repeated across spectral resolutions. Inset values give the pairwise Pearson correlation coefficients for proportion key words correct across spectral resolutions.

Figure 1 shows each participant's PRESTO sentence recognition accuracy across levels of vocoder spectral resolution. As expected, performance was driven by spectral resolution, with mean proportion key words correct of 88.1%, 77.1%, and 39.1% for 16-, 8-, and 4-channel spectral resolutions, respectively. Individual performance relative to the group was consistent across spectral resolutions, which is reflected in the strong cross-resolution correlation coefficients shown in the inset of Figure 1. The strength of these correlations was not diminished by floor or ceiling performance for any resolution. The distribution of data was also similar across resolutions, as shown by similar standard deviation of key words correct (5.6%, 6.4%, and 7.2% for 16-, eight-, and four-channel resolutions, respectively).

We analyzed these data in two ways. First, as planned, we used speech recognition accuracy at each vocoder level as the dependent variable in subsequent analyses. Second, because accuracy was highly correlated across spectral resolutions, we made the post hoc decision to combine performance across spectral resolutions into a single composite sentence recognition score via principal components analysis. Table 1 shows the results of this analysis. The first component had an eigenvalue greater than 1 and explained most of the variance. The second and third components had small eigenvalues and were judged to be too small to consider further. The first component loaded equally across resolutions, demonstrating that individual performance relative to the population was highly consistent across spectral resolutions. Therefore, individual scores on the first component produced by this principal components analysis were used as a composite estimate of PRESTO sentence recognition ability.

**Table 1.** Component loading and variance explained in Perceptually Robust English Sentence Test Open-set (PRESTO) sentence recognition accuracy across spectral resolutions.

No. channels	Component		
	1	2	3
16	****0.57****	−0.68	−0.47
8	****0.59****	−0.05	0.81
4	****0.57****	0.74	−0.37
Eigenvalue	****2.43****	0.34	0.23
Variance explained	**81%**	11%	8%

*Note.* The component with an eigenvalue of greater than 1 is in bold.

### Predictor 1: Fluid Intelligence

**Figure 2.**
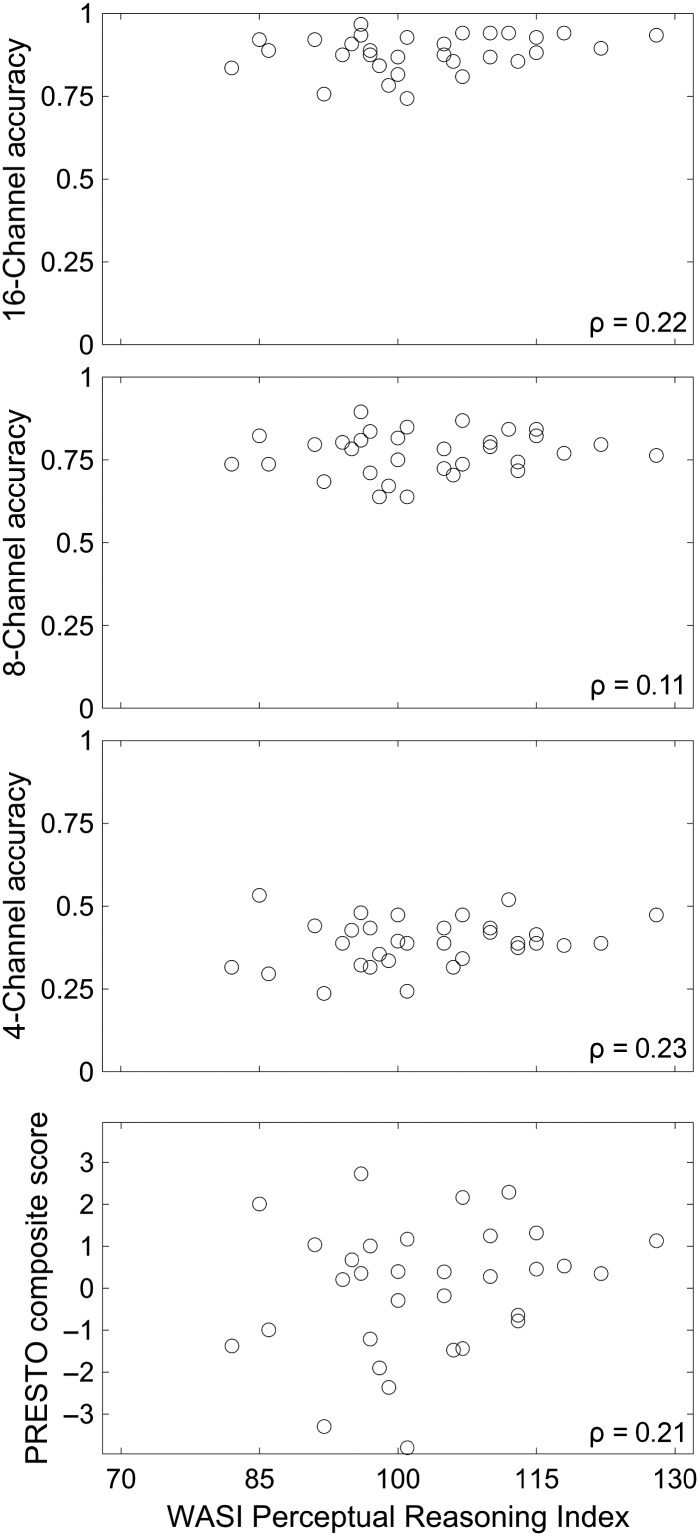
Relationship between fluid intelligence (Wechsler Abbreviated Scale of Intelligence [WASI] Perceptual Reasoning Index) and sentence recognition accuracy at each spectral resolution (16, eight, and four channels, proportion correct) and aggregated across spectral resolutions (Perceptually Robust English Sentence Test Open-set [PRESTO] composite score, normed score). Each point represents one individual. Correlation coefficients were not statistically significant at *p* < .05.

The Perceptual Reasoning Index of the WASI was calculated by summing scores on the Block Design and Matrix Reasoning subtests and converting the sum to normative values (Wechsler, 2011). The Perceptual Reasoning Index had a mean of 103.0, an *SD* of 10.8, and maximum and minimum values of 82 and 128, respectively. These statistics indicate that the distribution of the index across our participants was positively skewed relative to the normative sample, which has a mean of 100 and an *SD* of 15. Figure 2 shows that the relationship between the Perceptual Reasoning Index and PRESTO sentence recognition accuracy was not statistically significant.

### Predictor 2: Serial Recall

**Figure 3.**
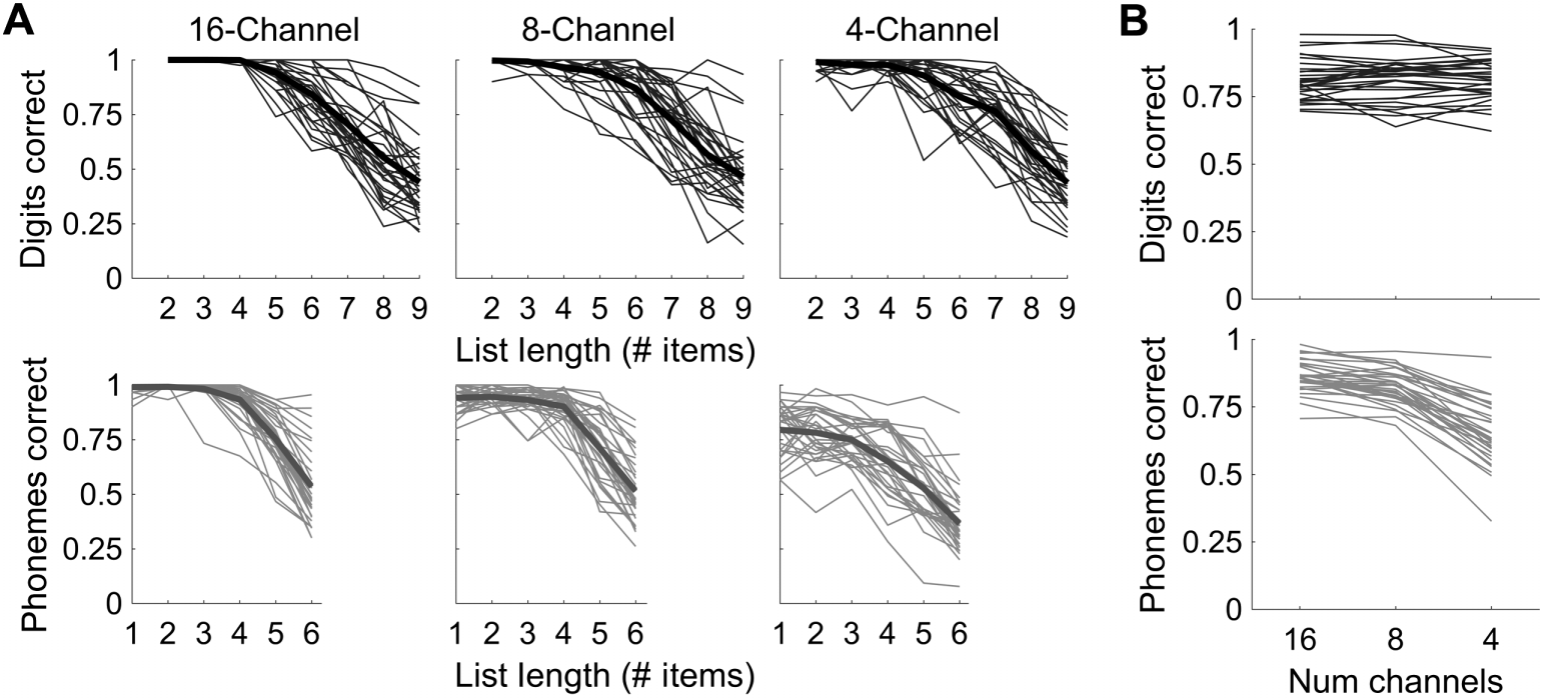
Digit and word serial recall performance across spectral resolutions. (A) Each thin line represents the proportion of digits or phonemes correctly recalled in the correct position for each list length by one individual, and thick lines show average recall rate across individuals for each list length. Digit span data are in the top row, and word span data are in the bottom row. (B) Each line represents the proportion of digits or phonemes correctly recalled across all list lengths, for each spectral resolution.

Figure 3A shows individual performance and group means for digit and word serial recall for each spectral resolution across the tested list lengths. Performance on digit and word serial recall was evaluated by counting the proportion of repeated digits and phonemes, which matched the item in the corresponding position in the target list. Proportional list scoring was used because it has good internal consistency (Conway et al., 2005) and provides more information about performance on a trial-by-trial basis than whole list scoring. Word lists were scored by the number of correct phonemes rather than the number of correct words to provide credit for partially correct words.

Figure 3B shows individual trends in mean proportional items correct across all list lengths for each spectral resolution. As shown, performance in digit serial recall was insensitive to spectral resolution, whereas word serial recall performance decreased as the spectral resolution decreased. This trend was confirmed with linear mixed-effects models, in which mean proportion correct was predicted by the number of channels as a categorical fixed effect and participant as a random effect (see Table 2). Performance was effectively identical for digit serial recall (group mean of 81% correct regardless of number of channels). Word serial recall significantly decreased with a decreasing number of channels (group mean of 86% correct in the 16-channel resolution; 82% in the 8-channel resolution, *t*(93) = −3.2, *p* = 1.7 × 10^−3^; and 65% in the 4-channel resolution, *t*(93) = −17.7, *p* = 1.3 × 10^−31^). Alternate scoring methods (Woods et al., 2011) produced similar trends (see Supplemental Material S1).

**Figure 4.**
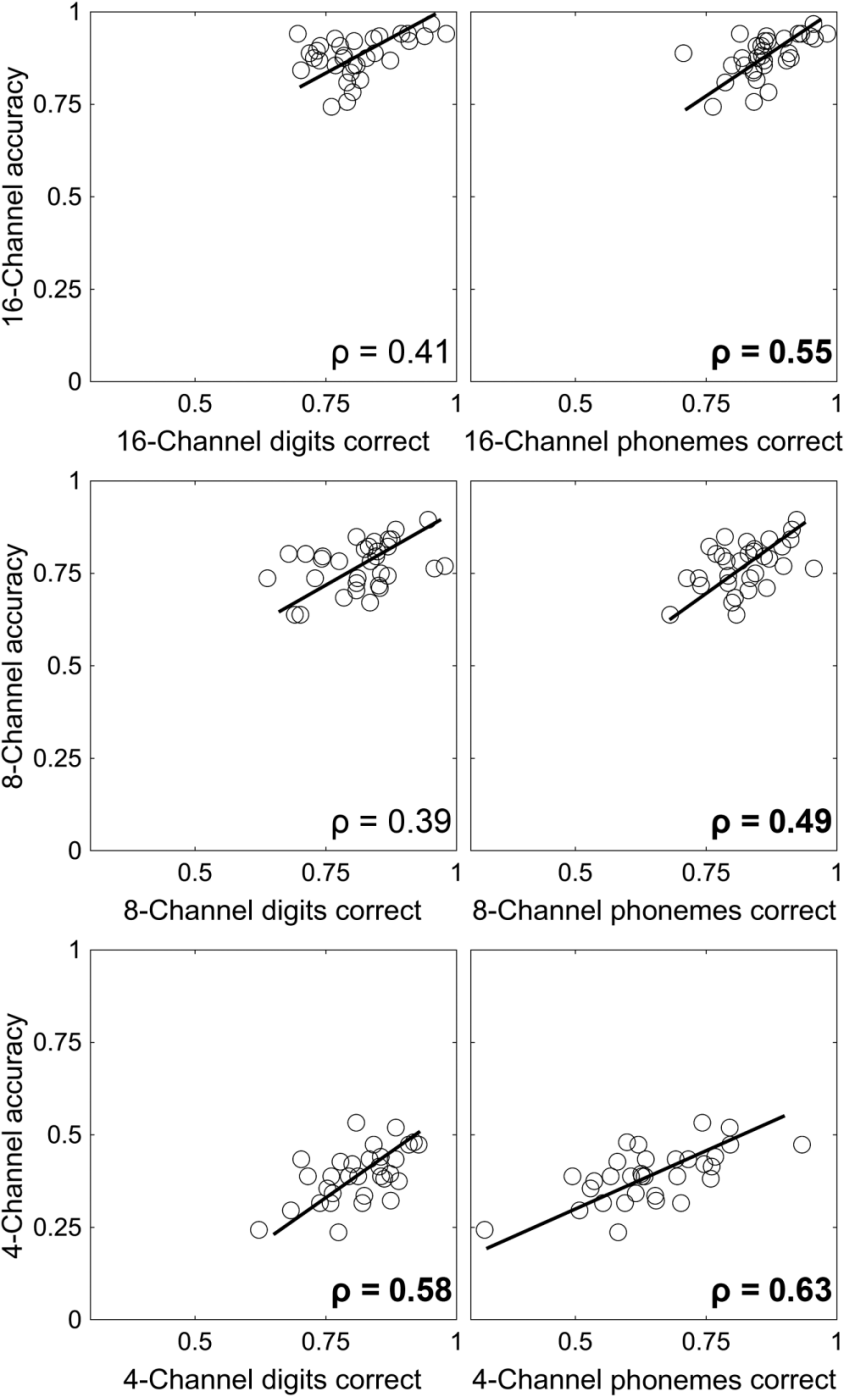
Relationship between serial recall and sentence recognition accuracy at each spectral resolution. Perceptually Robust English Sentence Test Open-set (PRESTO) accuracy at each spectral resolution is shown as a function of the proportion of digits correct (left column) and phonemes correct (right column) in the serial recall task in the corresponding spectral resolution. In all panels, each point represents one individual, and lines show the standard major axis regression fit (Legendre, 2013) across individuals. Correlation coefficients were all significant at *p* < .05, and values in bold were significant after Bonferroni correction for multiple comparisons (*p* < .0083).

Figure 4 shows the relationship between serial recall accuracy and PRESTO sentence recognition accuracy in matched spectral resolutions. As shown, significant correlations were observed in each spectral resolution between PRESTO accuracy and both digit serial recall and word serial recall, although the correlations for serial recall accuracy for digits at the 16- and 8-channel resolutions were not significant after correcting for multiple comparisons.

**Figure 5.**
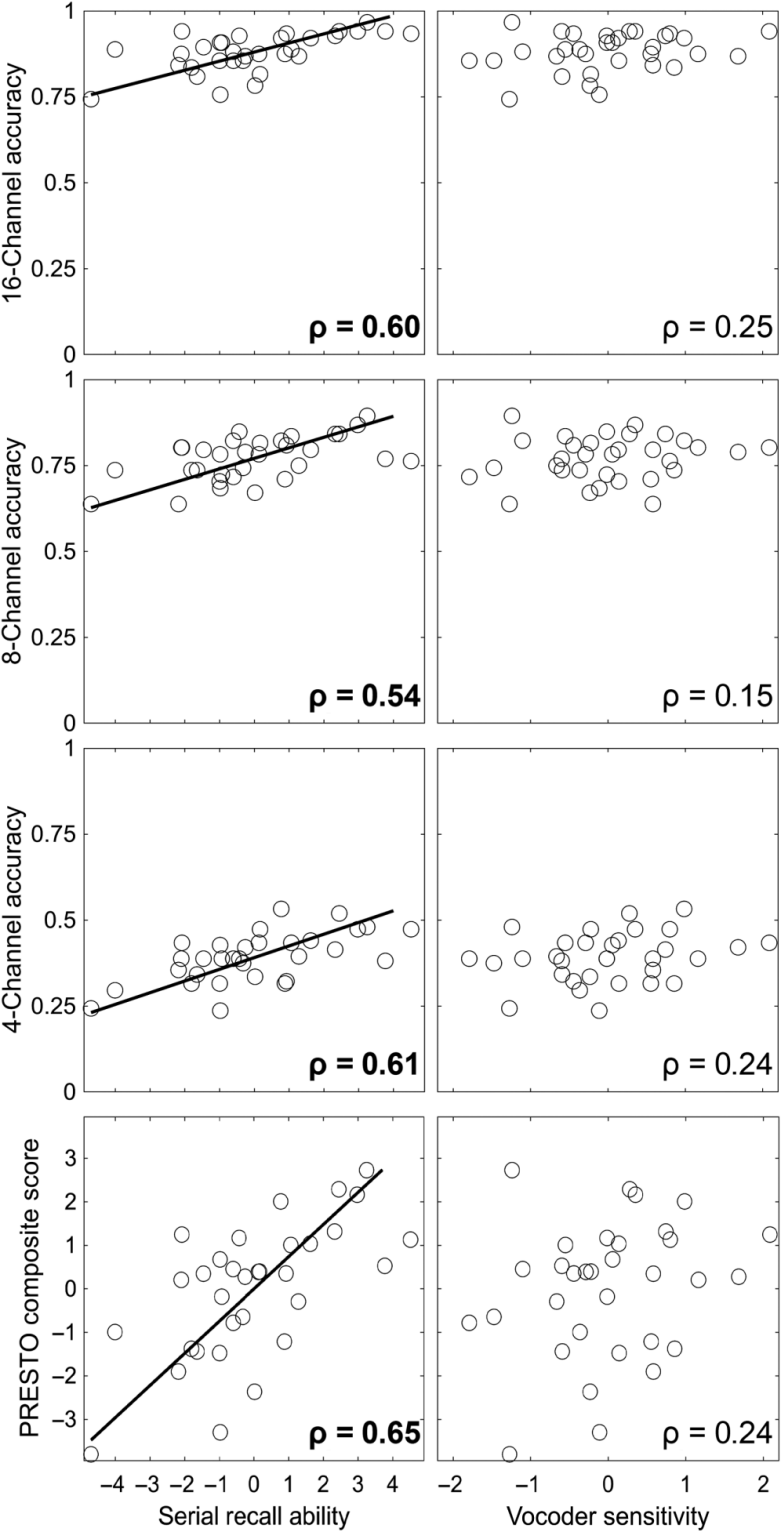
Relationship between serial recall principal components and Perceptually Robust English Sentence Test Open-set (PRESTO sentence recognition accuracy at each spectral resolution (16, eight, and four channels, proportion correct) and aggregated across spectral resolutions (PRESTO composite score, normed score). Serial recall ability (left column) reflects individual scores along the first principal component in Table 2, and vocoder sensitivity (right column) reflects individual scores along the second principal component. Correlation coefficients in bold were significant after Bonferroni correction for multiple comparisons (*p* < .0063). Each point represents one individual, and lines show standard major axis regression fits for significant correlations.

Table 2 shows the results of principal components analysis for serial recall performance across stimuli and spectral resolutions. This analysis produced similar results as in the study of Bosen and Luckasen (2019). Component 1 is loaded roughly equally across all combinations of stimulus and spectral resolution and therefore reflects overall serial recall ability. Component 2 loaded in different directions for digit and word stimuli, and reflects sensitivity to vocoding for word stimuli. However, Component 2 was smaller than expected based on previous findings. Specifically, it had an eigenvalue of less than 1, which would normally make it too small to consider if we did not have these previous findings to support its inclusion in subsequent analysis. The remaining components were judged too small to consider further. These components were used as predictors of PRESTO sentence recognition accuracy, as shown in Figure 5. Serial recall ability (Component 1) was a significant predictor of PRESTO sentence recognition at all spectral resolutions and for the composite score, whereas vocoder sensitivity (Component 2) was not significantly correlated with PRESTO sentence recognition in any condition. In addition to these planned analyses, a post hoc simple linear correlation found a marginal correlation between serial recall ability and the WASI Perceptual Reasoning Index (ρ = 0.35, *p* = .052).

**Table 2.** Component loading and variance explained in digit and word serial recall accuracy across stimuli and spectral resolutions.

Stimuli	No. channels	Component					
		1	2	3	4	5	6
Digits	16	**0.41**	−0.35	−0.13	0.78	−0.05	−0.29
	8	**0.41**	−0.45	−0.36	−0.14	−0.28	0.63
	4	**0.43**	−0.27	−0.21	−0.43	0.57	−0.44
Words	16	**0.43**	0.08	0.60	−0.39	−0.51	−0.18
	8	**0.41**	0.45	0.33	0.15	0.51	0.50
	4	**0.36**	0.63	−0.58	0.09	−0.28	−0.21
Eigenvalue		**4.44**	0.79	0.29	0.22	0.17	0.10
Variance explained		**74%**	13%	5%	3%	3%	2%

*Note.* The component with an eigenvalue of greater than 1 is in bold.

### Predictor 3: Attention

Accuracy on the color–shape categorization and Stroop tasks was at or near ceiling for all participants, with median values and ranges of 98.75% (ranging from 86.25% to 100%) and 97.5% (ranging from 88.75% to 100%), respectively. Incorrect trials were discarded from reaction time analysis. Outlier reaction times were identified as in the study of Schmiedek et al. (2007). Reaction times greater than 4 *SD*s above the mean was labeled as an outlier and removed, and this process was repeated until no remaining reaction times were labeled as outliers. This definition of outliers removed at most seven trials from one condition in the color–shape categorization task, so sufficient data were retained for reaction time distribution analyses.

**Figure 6.**
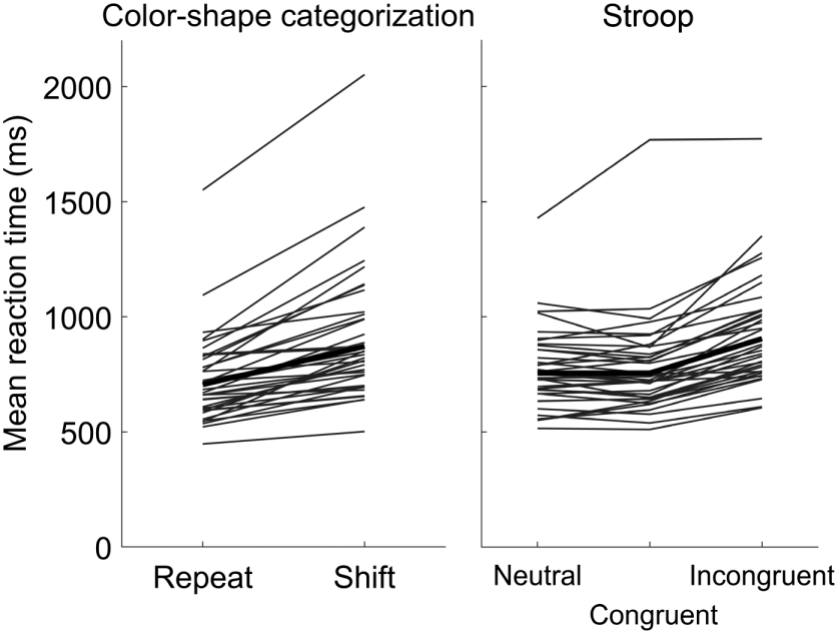
Mean reaction times for the color–shape categorization (left) and Stroop (right) tasks. Thin lines represent the geometric mean reaction time for an individual across conditions, and thick lines show the group geometric mean reaction time in each condition.

Figure 6 shows mean reaction times for the color–shape categorization and Stroop tasks. In the color–shape categorization task, average reaction times were faster when the categorization cue was repeated from the previous trial than when it switched. This trend was evident in all participants, with a significant median difference of 145 ms between the geometric mean reaction time in each condition (Wilcoxon signed-ranks test, Z = 5.37, *p* < .001). Similar slowing was evident in the incongruent condition of the Stroop task, which had median differences of 153 and 137 ms relative to the neutral and congruent conditions, respectively (Z = 5.37, *p* < .001 in both comparisons). Neutral and congruent conditions were not significantly different from one another (Z = 0.41, *p* = .68). Confirmation of these expected effects enabled the planned comparison of reaction time differences with speech recognition.

Table 3 provides correlation coefficients for color–shape categorization, Stroop, and TOVA task outcomes with sentence recognition. For color–shape categorization and Stroop tasks, metrics were the reaction time differences across conditions as described above. For the TOVA, four comparisons to normative samples are calculated by the TOVA software and reflect overall speed (Response Time CNS), variability in response time (Variability CNS), the number of commission errors made (Commission CNS), and the number of omission errors made (Omission CNS). As shown, no correlations were significant, even before correcting for multiple comparisons.

**Table 3.** Correlation coefficients between attention task outcome measures (color–shape categorization and Stroop mean differences and Test of Variables of Attention [TOVA] comparisons to normative samples [CNS]) and Perceptually Robust English Sentence Test Open-set (PRESTO) sentence recognition accuracy across spectral resolutions.

Attention metric	PRESTO, no. channels			
	16	8	4	Composite
Color–shape categorization RT difference	−.08	−.11	−.17	−.14
Stroop incongruent–congruent RT difference	−.16	.02	.05	−.03
Stroop incongruent–neutral RT difference	−.19	−.07	−.11	−.13
TOVA Response Time CNS	.03	.07	−.20	−.04
TOVA Variability CNS	.09	.12	−.14	.03
TOVA Commission CNS	.19	.25	.22	.24
TOVA Omission CNS	−.07	.12	−.05	.00

*Note.* No correlations were significant at the *p* < .05 level. RT = reaction time.

In addition to the planned analyses, we also tested whether these attention measures were correlated with the WASI Perceptual Reasoning Index and the two serial recall principal components post hoc, which is shown in Table 4. No significant correlations were observed.

**Table 4.** Correlation coefficients between attention task outcome measures as in Table 3 and the Wechsler Abbreviated Scale of Intelligence (WASI) Perceptual Reasoning Index (PRI) and serial recall principal components.

Attention metric	WASI PRI	Serial recall ability	Vocoder sensitivity
Color–shape categorization RT difference	−.21	−.24	−.10
Stroop incongruent–congruent RT difference	−.21	−.09	.21
Stroop incongruent–neutral RT difference	−.30	−.30	.17
TOVA Response Time CNS	−.32	−.12	.10
TOVA Variability CNS	−.18	.04	−.01
TOVA Commission CNS	.22	.12	−.11
TOVA Omission CNS	−.09	−.17	−.13

*Note.* No correlations were significant at the *p* < .05 level. RT = reaction time; TOVA = Test of Variables of Attention; CNS = comparisons to normative samples.

Given the apparent lack of relationship between attention measures and any other variable of interest in this study, we conducted a post hoc analysis on the reaction time data to determine if an aspect of reaction time other than cross-condition differences in the color–shape categorization and Stroop tasks was associated with the other variables of interest. Exponential Gaussian functions were fit to reaction time distributions in each condition for each individual (see Supplemental Material S1 for examples). This function has three free parameters, which describe the mean and variance of the Gaussian component (μ and σ, respectively) and the size of the long tail (τ). Previous research has indicated that individual variability in τ is associated with working memory and reasoning (Schmiedek et al., 2007), which is why we looked for similar relationships here. Table 5 shows the correlation of mean reaction time and the exponential Gaussian fit parameters, averaged across all conditions of both the color–shape categorization and Stroop tasks. Here, a stronger correlation emerged between overall reaction time and the WASI Perceptual Reasoning Index, although these correlations did not appear to be specific to a particular component of the reaction time distributions. There was also a marginal correlation between mean reaction time and serial recall ability (*p* = .04), which arises from correlations between τ and serial recall ability (*p* = .03).

**Table 5.** Correlation coefficients between mean reaction time fit parameters across all color–shape categorization and Stroop task conditions and the variables of interest in Tables 3 and 4. PRESTO = Perceptually Robust English Sentence Test Open-set.

Variable	PRESTO, no. channels				WASI PRI	Serial recall ability	Vocoder sensitivity
	16	8	4	Composite			
Mean RT	−.06	−.10	−.18	−.13	−.46*	−.37*	.10
Mean μ	.06	−.06	−.07	−.03	−.37*	−.22	.21
Mean σ	−.14	−.13	−.07	−.13	−.55*	−.26	−.02
Mean τ	−.10	−.11	−.20	−.15	−.43*	−.38*	.02

*Note.* Asterisks indicate correlations that were significant at *p* < .05, and the bold value for the mean σ–WASI correlation indicates significance after correcting for multiple comparisons (*p* = 4.5 × 10^−4^ with a threshold of 1.8 × 10^−3^ after Bonferroni correction). WASI PRI = Wechsler Abbreviated Scale of Intelligence Perceptual Reasoning Index; RT = reaction time.

## Discussion

Individual ability to repeat vocoded PRESTO sentences was consistent relative to the group across spectral resolutions, even for the easiest (16-channel) condition. This consistency led us to conduct a post hoc dimensionality reduction to describe performance across spectral resolutions as a single composite score, as summarized in Table 1. This consistent performance across spectral resolutions contradicts our original hypothesis that cognitive factors should play a larger role in harder listening conditions.

This individual consistency across spectral resolutions indicates that PRESTO sentences are good for testing speech recognition for two reasons. First, individual consistency across listening conditions is desirable if this finding extends to listeners with hearing loss, because we cannot control the quality of auditory input these listeners receive. Instead, it should be possible to estimate each individual's auditory quality through psychophysical testing and then factor out the effect of auditory quality on sentence recognition to obtain an estimate of individual differences in sentence recognition associated with nonauditory factors. Second, the presence of individual variability in the 16-channel condition indicates that even individuals with hearing loss who have relatively good quality auditory input will still exhibit variability in sentence recognition. This is desirable, because it is unlikely that there is a threshold of quality auditory input over which sentence recognition accuracy will reach 100%. If there were, this threshold would minimize individual differences in speech recognition for individuals with auditory quality above that threshold, which would mask the role of nonauditory factors in speech recognition for those listeners. Overall, our speech recognition results are in agreement with previous findings that, while auditory quality has the largest impact on sentence recognition, cognitive ability also plays a substantial role in individual variability in sentence recognition (Akeroyd, 2008).

The nonauditory variability in vocoded sentence recognition was primarily explained by individual variability in serial recall ability (see Figure 5). The strength of this relationship was stronger than typically observed between working memory tasks and speech recognition in noise (Dryden et al., 2017), even for working memory tasks that use auditory stimuli (S. L. Smith et al., 2016). There are several theoretical explanations that could account for this relationship. First, stimuli in both the serial recall and sentence recognition tasks were aurally presented. This common sensory pathway facilitates the use of auditory-specific storage and rehearsal processes (Kane et al., 2004), which is often conceptualized as the phonological loop (Baddeley, 2012). Additionally, avoiding the need to map stimuli from a visual orthographic representation, as is typical in commonly used complex span tasks (Conway et al., 2005), eliminates this potential source of individual variability. Second, both tasks required a verbal response. This common response method relies on redintegration and production. Redintegration refers to the ability to restore decayed short-term memory traces (Hulme et al., 1997; T. Jones & Farrell, 2018), which could be alternatively characterized as retrieval from secondary memory (Unsworth & Engle, 2007b) or top-down restoration of speech (Benard et al., 2014). Language production mechanisms have also been posited to underlie verbal serial recall (Acheson & Macdonald, 2009a, 2009b; Maidment & Macken, 2012) and would be supported by the auditory–motor connection in Hickok and Poeppel's (2004) dual stream framework. Third, both tasks may invoke common linguistic experience. In addition to short-term memory, serial span tasks are influenced by linguistic experience. This is evident in lexical frequency and neighborhood density effects in serial recall (Roodenrys et al., 2002) and has been demonstrated even in digit span (G. Jones & Macken, 2015). Given that vocabulary measures are also correlated with individual differences in recognition of accented speech and in noise (Bent et al., 2016; McLaughlin et al., 2018), part of the link between serial recall and sentence recognition we observed here is likely explained by individual variability in linguistic experience. Fourth, both tasks presented stimuli at a fairly rapid rate. In complex span tasks, the time between items to be remembered is often much longer than in serial recall or sentence recognition tasks, which facilitates covert retrieval between memoranda (McCabe, 2008) in a manner that would be less pronounced in a serial recall task.

The common loading of digit span and word span onto a single overall measure of serial recall ability (see Table 2, Component 1) agrees with our previous findings (Bosen & Luckasen, 2019). This common loading indicates that digit span can be used to estimate serial recall ability across a range of spectral resolutions. This finding is important for subsequent research in listeners with hearing loss, because it indicates that degraded spectral resolution from hearing loss should not limit our ability to estimate verbal serial recall ability. Individual sensitivity to vocoding (see Table 2, Component 2) was not significantly correlated with sentence recognition. This is surprising, given that both word span and key words in PRESTO sentences were drawn from a larger set of words. This finding should be interpreted with caution, because the vocoder sensitivity component was relatively small, which means estimates of its value across individuals are imprecise. Word span performance was a stronger predictor of sentence recognition accuracy than digit span performance, which indicates that the ability to recognize and store words drawn from a larger set is an important component of sentence recognition, just not one that was well represented in this principal components model. The fact that serial recall ability was the better predictor of sentence recognition underscores the idea that meaningful sentences are more than just a sequence of words.

The WASI Perceptual Reasoning Index was marginally correlated with serial recall but was not correlated with sentence recognition (see Figure 2). The sampled distribution of the index was positively skewed relative to the normative sample, which is likely because our participants were predominantly recruited from a nearby college. Positive skew diminishes the range of performance relative to a normative sample, which would make it more difficult to detect a relationship with other measures. Previous research indicates that this relationship should exist, and our sample size was insufficient to provide definitive evidence for it. The observed correlation between the WASI Perceptual Reasoning Index and overall serial span ability is similar to the previously observed correlations between short-term memory and general fluid intelligence latent constructs (Gignac & Weiss, 2015; Unsworth & Engle, 2007a). There was a nonsignificant trend relating the WASI Perceptual Reasoning Index to sentence recognition, which had approximately the same effect size as previously reported relationships between other tests of fluid intelligence and vocoded speech (O'Neill et al., 2019). Previous research has found weak correlations between speech recognition and various cognitive subscales (Dryden et al., 2017), which may reflect the general association between fluid intelligence and speech recognition. Our results suggest that serial recall is a more proximal test of the skills necessary to perform vocoded sentence recognition.

The planned attentional measures were not predictive of sentence recognition (see Table 3) or serial recall (see Table 4). Therefore, our results suggest that individual differences in vocoded sentence recognition accuracy are primarily associated with short-term memory. Stroop task performance has sometimes been shown to predict speech in noise in older adults (Janse, 2012, although see Knight & Heinrich, 2017), so it is possible that our lack of correlation may have been a result of testing young adults and using vocoding, rather than speech in noise, to degrade speech. Additionally, the lack of attentional association may be because attention task performance is driven by multiple factors. Specifically, performance on the Stroop task has been previously demonstrated to be determined by goal maintenance and competition resolution (Kane & Engle, 2003). Dissociating these factors is necessary to evaluate whether they differentially contribute to speech recognition and whether their contributions change with age. Finally, individual differences in attentional control, particularly in tasks that involve conflicting information, may not be characterizable from mean cross-condition reaction time differences and instead require model fitting to reaction time distributions (White et al., 2016, 2018). Even with more nuanced approaches, the idea that inhibition is a valid psychometric construct has recently been called into question (Rey-Mermet et al., 2018), so caution is required when using attentional mechanisms to explain variability in speech recognition. Post hoc analysis of reaction time distributions across color–shape categorization and Stroop tasks found correlations between the WASI Perceptual Reasoning Index and reaction time and a marginal correlation between the reaction time distribution tail parameter τ and serial recall ability (see Table 5). These correlations are weak, and this analysis was unplanned, so these results should be interpreted with caution but are consistent with the idea that overall processing speed may be a more meaningful metric of cognitive ability than cross-condition reaction time differences.

Additional work is necessary to confirm and elaborate on our findings. While we observed a moderate correlation between serial recall and vocoded sentence recognition, the relatively small sample size provides a wide confidence interval for this estimate, so the true effect size likely differs from the estimate reported here. The sample size may also hide weak correlations that are present across tasks. The observed effect may also not generalize across different populations and speech tasks. As described in the introduction, differences in hearing status and aging would likely alter the apparent strength of these correlations, as well as introduce new sources of variability that are not significant factors in young adults with normal hearing. Our use of solely young adults with normal hearing allowed us to meaningfully compare sentence recognition across listening conditions but does not permit extrapolation of the observed relationships to individuals with hearing loss or who differ in age from the current sample. Because our speech task had a single vocoded stream with no competing streams, the role of attention was likely minimized. The cognitive abilities that facilitate speech recognition depend on the speech task being performed (Heinrich et al., 2015; McLaughlin et al., 2018). For example, our lack of a correlation between attention and sentence recognition differs from speech in noise results from the study of Heinrich et al. (2015). Given that our stimuli were vocoded, not presented in noise as in their study, it is possible that segregating target speech from a background masker in their study placed additional attentional demands on their participants, which were not present in our study. Comparing predictors of recognition across speech in noise and vocoded speech would likely distinguish stimulus-specific and general relationships between cognition and speech recognition. Along those lines, speech comprehension tasks, such as those described by Best et al. (2016), Daneman and Merikle (1996), and Xia et al. (2017), likely rely on a different set of cognitive factors than speech recognition tasks and might be more useful for assessing skills related to quality of life. Finally, although serial recall was a strong predictor of sentence recognition, it is not a pure measure of one latent construct of memory, but rather reflects a mixture of abilities (Unsworth & Engle, 2006). Thus, although the correlation we observed is stronger than previously observed relationships, the problem remains of identifying the latent constructs that underlie this correlation.

## Supplementary Material

10.1044/2020_JSLHR-19-00319SMS1 Supplemental Material S1 A complete set of words and word lists.

## References

[bib1] Acheson D. J., & Macdonald M. C. (2009a). Twisting tongues and memories: Explorations of the relationship between language production and verbal working memory. Journal of Memory and Language, 60(3), 329–350. https://doi.org/10.1016/j.jml.2008.12.002 21165150 10.1016/j.jml.2008.12.002. PMC3001594

[bib2] Acheson D. J., & Macdonald M. C. (2009b). Verbal working memory and language production: Common approaches to the serial ordering of verbal information. Psychological Bulletin, 135(1), 50–68. https://doi.org/10.1037/a0014411 19210053 10.1037/a0014411 PMC3000524

[bib3] Akeroyd M. A. (2008). Are Individual differences in speech reception related to individual differences in cognitive ability? A survey of twenty experimental studies with normal and hearing-impaired adults. International Journal of Audiology, 47(Suppl. 2), S53–S71. https://doi.org/10.1080/14992020802301142 19012113 10.1080/14992020802301142

[bib4] Arlinger S., Lunner T., Lyxell B., & Pichora-Fuller M. K. (2009). The emergence of cognitive hearing science. Scandinavian Journal of Psychology, 50(5), 371–384. https://doi.org/10.1111/j.1467-9450.2009.00753.x 19778385 10.1111/j.1467-9450.2009.00753.x

[bib5] Baddeley A. (2012). Working memory: Theories, models, and controversies. Annual Review of Psychology, 63, 1–29. https://doi.org/10.1146/annurev-psych-120710-100422 10.1146/annurev-psych-120710-100422 21961947

[bib6] Baldwin C. L., & Ash I. K. (2011). Impact of sensory acuity on auditory working memory span in young and old adults. Psychology of Aging, 26(1), 85–91. https://doi.org/10.1037/a0020360 10.1037/a0020360 PMC3062694 20718539

[bib7] Barrouillet P., Bernardin S., & Camos V. (2004). Time constraints and resource sharing in adults' working memory spans. Journal of Experimental Psychology: General, 133(1), 83–100. https://doi.org/10.1037/0096-3445.133.1.83 14979753 10.1037/0096-3445.133.1.83

[bib8] Barrouillet P., Bernardin S., Portrat S., Vergauwe E., & Camos V. (2007). Time and cognitive load in working memory. Journal of Experimental Psychology: Learning, Memory, and Cognition, 33(3), 570–585. https://doi.org/10.1037/0278-7393.33.3.570 10.1037/0278-7393.33.3.570 17470006

[bib9] Benard M. R., Mensink J. S., & Başkent D. (2014). Individual differences in top-down restoration of interrupted speech: Links to linguistic and cognitive abilities. The Journal of the Acoustical Society of America, 135(2), EL88–EL94. https://doi.org/10.1121/1.4862879 25234920 10.1121/1.4862879

[bib10] Bent T., Baese-Berk M., Borrie S. A., & McKee M. (2016). Individual differences in the perception of regional, nonnative, and disordered speech varieties. The Journal of the Acoustical Society of America, 140(5), 3775–3386. https://doi.org/10.1121/1.4966677 27908060 10.1121/1.4966677

[bib11] Best V., Streeter T., Roverud E., Mason C. R., & Kidd G. Jr. (2016). A flexible question-and-answer task for measuring speech understanding. Trends in Hearing, 20, 1–8. https://doi.org/10.1177/2331216516678706 10.1177/2331216516678706 PMC5131808 27888257

[bib12] Bosen A. K., & Chatterjee M. (2016). Band importance functions of listeners with cochlear implants using clinical maps. The Journal of the Acoustical Society of America, 140(5), 3718–3727. https://doi.org/10.1121/1.4967298 27908046 10.1121/1.4967298 PMC5392084

[bib13] Bosen A. K., & Luckasen M. C. (2019). Interactions between item set and vocoding in serial recall. Ear and Hearing, 40(6), 1404–1417. https://doi.org/10.1097/AUD.0000000000000718 31033634 10.1097/AUD.0000000000000718 PMC6776730

[bib14] Brysbaert M., & New B. (2009). Moving beyond Kučera and Francis: A critical evaluation of current word frequency norms and the introduction of a new and improved word frequency measure for American English. Behavior Research Methods, 41(4), 977–990. https://doi.org/10.3758/BRM.41.4.977 19897807 10.3758/BRM.41.4.977

[bib15] Chen Z., & Cowan N. (2009). How verbal memory loads consume attention. Memory and Cognition, 37(6), 829–836. https://doi.org/10.3758/MC.37.6.829 19679862 10.3758/MC.37.6.829 PMC2804027

[bib16] Colom R., Privado J., García L. F., Estrada E., Cuevas L., & Shih P. C. (2015). Fluid intelligence and working memory capacity: Is the time for working on intelligence problems relevant for explaining their large relationship? Personality and Individual Differences, 79, 75–80. https://doi.org/10.1016/j.paid.2015.01.051

[bib17] Conway A. R. A., Kane M. J., Bunting M. F., Hambrick D. Z., Wilhelm O., & Engle R. W. (2005). Working memory span tasks: A methodological review and user's guide. Psychonomic Bulletin and Review, 12(5), 769–786. https://doi.org/10.3758/BF03196772 16523997 10.3758/bf03196772

[bib18] Conway A. R. A., & Kovacs K. (2013). Individual differences in intelligence and working memory: A review of latent variable models *.* Psychology of Learning and Motivation—Advances in Research and Theory, 58, 233–270. https://doi.org/10.1016/B978-0-12-407237-4.00007-4

[bib19] Cowan N. (2001). The magical number 4 in short term memory: A reconsideration of storage capacity. Behavioral and Brain Sciences, 24(1), 87–186. https://doi.org/10.1017/S0140525X01003922 11515286 10.1017/s0140525x01003922

[bib20] Daneman M., & Carpenter P. A. (1980). Individual differences in working memory and reading. Journal of Verbal Learning, 19(4), 450–466. https://doi.org/10.1016/S0022-5371(80)90312-6

[bib21] Daneman M., & Merikle P. M. (1996). Working memory and language comprehension: A meta-analysis. Psychonomic Bulletin and Review, 3(4), 422–433. https://doi.org/10.3758/BF03214546 24213976 10.3758/BF03214546

[bib22] Diamond A. (2013). Executive functions. Annual Review of Psychology, 64(1), 135–168. https://doi.org/10.1146/annurev-psych-113011-143750 10.1146/annurev-psych-113011-143750 PMC4084861 23020641

[bib23] DiNino M., Wright R. A., Winn M. B., & Arenberg Bierer J. (2016). Vowel and consonant confusions from spectrally manipulated stimuli designed to simulate poor cochlear implant electrode-neuron interfaces. The Journal of the Acoustical Society of America, 140(6), 4404–4418. https://doi.org/10.1121/1.4971420 28039993 10.1121/1.4971420 PMC5392103

[bib24] Dryden A., Allen H. A., Henshaw H., & Heinrich A. (2017). The association between cognitive performance and speech-in-noise perception for adult listeners: A systematic literature review and meta-analysis. Trends in Hearing, 21, 1–21. https://doi.org/10.1177/2331216517744675 10.1177/2331216517744675 PMC5734454 29237334

[bib25] Dupuis K., Pichora-Fuller M. K., Chasteen A. L., Marchuk V., Singh G., & Smith S. L. (2015). Effects of hearing and vision impairments on the Montreal Cognitive Assessment. Aging, Neuropsychology, and Cognition, 22(4), 413–437. https://doi.org/10.1080/13825585.2014.968084 10.1080/13825585.2014.968084 25325767

[bib26] Emery L., Hale S., & Myerson J. (2008). Age differences in proactive interference, working memory, and abstract reasoning. Psychology and Aging, 23(3), 634–645. https://doi.org/10.1037/a0012577 18808252 10.1037/a0012577 PMC2556888

[bib27] Engle R. W., & Kane M. J. (2004). Executive attention, working memory capacity, and a two-factor theory of cognitive control. The Psychology of Learning and Motivation, 44, 145–199. https://doi.org/10.1016/S0079-7421(03)44005-X

[bib28] Faulkner K. F., Tamati T. N., Gilbert J. L., & Pisoni D. B. (2015). List equivalency of PRESTO for the evaluation of speech recognition. Journal of the American Academy of Audiology, 26(6), 582–594. https://doi.org/10.3766/jaaa.14082 26134725 10.3766/jaaa.14082 PMC6205295

[bib29] Friedman N. P., Miyake A., Young S. E., Defries J. C., Corley R. P., & Hewitt J. K. (2008). Individual differences in executive functions are almost entirely genetic in origin. Journal of Experimental Psychology, 137(2), 201–225. https://doi.org/10.1037/0096-3445.137.2.201 18473654 10.1037/0096-3445.137.2.201 PMC2762790

[bib30] Friesen L. M., Shannon R. V., Baskent D., & Wang X. (2001). Speech recognition in noise as a function of the number of spectral channels: Comparison of acoustic hearing and cochlear implants. The Journal of the Acoustical Society of America, 110(2), 1150–1163. https://doi.org/10.1121/1.1381538 11519582 10.1121/1.1381538

[bib31] Gignac G. E. (2014). Fluid intelligence shares closer to 60% of its variance with working memory capacity and is a better indicator of general intelligence. Intelligence, 47, 122–133. https://doi.org/10.1016/j.intell.2014.09.004

[bib32] Gignac G. E., & Weiss L. G. (2015). Digit span is (mostly) related linearly to general intelligence: Every extra bit of span counts. Psychological Assessment, 27(4), 1312–1323. https://doi.org/10.1037/pas0000105 25774642 10.1037/pas0000105

[bib33] Gilbert J. L., Tamati T. N., & Pisoni D. B. (2013). Development, reliability, and validity of PRESTO: A new high-variability sentence recognition test. Journal of the American Academy of Audiology, 24(1), 26–36. https://doi.org/10.3766/jaaa.24.1.4 23231814 10.3766/jaaa.24.1.4 PMC3683852

[bib34] Gordon-Salant S., & Cole S. S. (2016). Effects of age and working memory capacity on speech recognition performance in noise among listeners with normal hearing. Ear and Hearing, 37(5), 593–602. https://doi.org/10.1097/AUD.0000000000000316 27232071 10.1097/AUD.0000000000000316

[bib106] Greenberg L. M. (2011). The Test of Variables of Attention (Version 8.0) [Computer software]. The ToVA Company.

[bib35] Greenwood D. D. (1990). A cochlear frequency-position function for several species–29 years later. The Journal of the Acoustical Society of America, 87(6), 2592–2605. https://doi.org/10.1121/1.399052 2373794 10.1121/1.399052

[bib36] Guerreiro M. J. S., & Van Gerven P. W. M. (2017). Disregarding hearing loss leads to overestimation of age-related cognitive decline. Neurobiology of Aging, 56, 180–189. https://doi.org/10.1016/j.neurobiolaging.2017.05.001 28559106 10.1016/j.neurobiolaging.2017.05.001

[bib37] Heinrich A., Henshaw H., & Ferguson M. A. (2015). The relationship of speech intelligibility with hearing sensitivity, cognition, and perceived hearing difficulties varies for different speech perception tests. Frontiers in Psychology, 6, 782 https://doi.org/10.3389/fpsyg.2015.00782 26136699 10.3389/fpsyg.2015.00782 PMC4468362

[bib38] Heinrich A., Henshaw H., & Ferguson M. A. (2016). Only behavioral but not self-report measures of speech perception correlate with cognitive abilities. Frontiers in Psychology, 7, 576 https://doi.org/10.3389/fpsyg.2016.00576 27242564 10.3389/fpsyg.2016.00576 PMC4876806

[bib39] Hickok G., & Poeppel G. (2004). Dorsal and ventral streams: A framework for understanding aspects of the functional anatomy of language. Cognition, 92(1–2), 67–99. https://doi.org/10.1016/j.cognition.2003.10.011 15037127 10.1016/j.cognition.2003.10.011

[bib40] Hulme C., Roodenrys S., Brown G. D. A., Schweickert R., Martin S., & Stuart G. (1997). Word-frequency effects on short-term memory tasks: Evidence for a redintegration process in immediate serial recall. Journal of Experimental Psychology: Learning Memory and Cognition, 23(5), 1217–1232. https://doi.org/10.1037/0278-7393.23.5.1217 10.1037//0278-7393.23.5.1217 9293631

[bib41] Humes L. E., Burk M. H., Coughlin M. P., Busey T. A., & Strauser L. E. (2007). Auditory speech recognition and visual text recognition in younger and older adults: Similarities and differences between modalities and the effects of presentation rate. Journal of Speech, Language, and Hearing Research, 50(2), 283–303. https://doi.org/10.1044/1092-4388(2007/021) 10.1044/1092-4388(2007/021) 17463230

[bib42] Humes L. E., Kidd G. R., & Lentz J. J. (2013). Auditory and cognitive factors underlying individual differences in aided speech-understanding among older adults. Frontiers in Systems Neuroscience, 7, 55 https://doi.org/10.3389/fnsys.2013.00055 24098273 10.3389/fnsys.2013.00055 PMC3787592

[bib43] Hunter C. R., & Pisoni D. B. (2018). Extrinsic cognitive load impairs spoken word recognition in high- and low-predictability sentences. Ear and Hearing, 39(2), 378–389. https://doi.org/10.1097/AUD.0000000000000493 28945658 10.1097/AUD.0000000000000493 PMC5821552

[bib44] Inquisit 5. (2016). [Computer software]. https://www.millisecond.com

[bib45] Janse E. (2012). A non-auditory measure of interference predicts distraction by competing speech in older adults. Aging, Neuropsychology, and Cognition, 19(6), 741–758. https://doi.org/10.1080/13825585.2011.652590 10.1080/13825585.2011.652590 22293017

[bib46] Jones G., & Macken B. (2015). Questioning short-term memory and its measurement: Why digit span measures long-term associative learning. Cognition, 144, 1–13. https://doi.org/10.1016/j.cognition.2015.07.009 26209910 10.1016/j.cognition.2015.07.009

[bib47] Jones T., & Farrell S. (2018). Does syntax bias serial order reconstruction of verbal short-term memory? Journal of Memory and Language, 100, 98–122. https://doi.org/10.1016/j.jml.2018.02.001

[bib48] Kaandorp M. W., Smits C., Merkus P., Festen J. M., & Goverts S. T. (2017). Lexical-access ability and cognitive predictors of speech recognition in noise in adult cochlear implant users. Trends in Hearing, 21, 1–15. https://doi.org/10.1177/2331216517743887 10.1177/2331216517743887 PMC5721962 29205095

[bib49] Kane M. J., & Engle R. W. (2003). Working-memory capacity and the control of attention: The contributions of goal neglect, response competition, and task set to Stroop interference. Journal of Experimental Psychology: General, 132(1), 47–70. https://doi.org/10.1037/0096-3445.132.1.47 12656297 10.1037/0096-3445.132.1.47

[bib50] Kane M. J., Hambrick D. Z., & Conway A. R. A. (2005). Working memory capacity and fluid intelligence are strongly related constructs: Comment on Ackerman, Beier, and Boyle (2005). Psychological Bulletin, 131(1), 66–71. https://doi.org/10.1037/0033-2909.131.1.66 15631552 10.1037/0033-2909.131.1.66

[bib51] Kane M. J., Hambrick D. Z., Tuholski S. W., Wilhelm O., Payne T. W., Engle R. W., & Randall W. (2004). The generality of working memory capacity: A latent-variable approach to verbal and visuospatial memory span and reasoning. Journal of Experimental Psychology: General, 133(2), 189–217. https://doi.org/10.1037/0096-3445.133.2.189 15149250 10.1037/0096-3445.133.2.189

[bib52] Knight S., & Heinrich A. (2017). Different measures of auditory and visual Stroop interference and their relationship to speech intelligibility in noise. Frontiers in Psychology, 8, 230 https://doi.org/10.3389/fpsyg.2017.00230 28367129 10.3389/fpsyg.2017.00230 PMC5355492

[bib53] Kronenberger W. G., Colson B. G., Henning S. C., & Pisoni D. B. (2014). Executive functioning and speech-language skills following long-term use of cochlear implants. Journal of Deaf Studies and Deaf Education, 19(4), 456–470. https://doi.org/10.1093/deafed/enu011 24903605 10.1093/deafed/enu011 PMC4146384

[bib54] Kronenberger W. G., Pisoni D. B., Henning S. C., & Colson B. G. (2013). Executive functioning skills in long-term cochlear implant users of cochlear implants: A case control study. Journal of Pediatric Psychology, 38(8), 902–914. https://doi.org/10.1093/jpepsy/jst034 23699747 10.1093/jpepsy/jst034 PMC3747713

[bib55] Leark R. A., Greenberg L. M., Kindschi C. L., Dupuy T. R., & Hughes S. J. (2008). TOVA professional manual. The TOVA Company.

[bib56] Legendre P. (2013). Model II regression user's guide, R Edition. R Vignette, 4, 1–14.

[bib57] Loughrey D. G., Kelly M. E., Kelley G. A., Brennan S., & Lawlor B. A. (2018). Association of age-related hearing loss with cognitive function, cognitive impairment, and dementia a systematic review and meta-analysis. JAMA Otolaryngology—Head & Neck Surgery, 144, 115–126. https://doi.org/10.1001/jamaoto.2017.2513 29222544 10.1001/jamaoto.2017.2513 PMC5824986

[bib58] Luce P. A., Feustel T. C., & Pisoni D. B. (1983). Capacity demands in short-term memory for synthetic and natural speech. Human Factors, 25(1), 17–32. https://doi.org/10.1177/001872088302500102 6840769 10.1177/001872088302500102 PMC3513698

[bib59] Lunner T. (2003). Cognitive function in relation to hearing aid use. International Journal of Audiology, 42(Suppl. 1), 49–58. https://doi.org/10.3109/14992020309074624 10.3109/14992020309074624 12918610

[bib60] Maidment D. W., & Macken W. J. (2012). The ineluctable modality of the audible: Perceptual determinants of auditory verbal short-term memory. Journal of Experimental Psychology: Human Perception and Performance, 38(4), 989–997. https://doi.org/10.1037/a0027884 22468724 10.1037/a0027884

[bib61] Mattys S. L., Davis M. H., Bradlow A. R., & Scott S. K. (2012). Speech recognition in adverse conditions: A review. Language and Cognitive Processes, 27(7–8), 953–978. https://doi.org/10.1080/01690965.2012.705006

[bib62] McCabe D. P. (2008). The role of covert retrieval in working memory span tasks: Evidence from delayed recall tests. Journal of Memory and Language, 58(2), 480–494. https://doi.org/10.1016/j.jml.2007.04.004 19633737 10.1016/j.jml.2007.04.004 PMC2715014

[bib63] McCabe D. P., Roediger H. L., McDaniel M. A., Balota D. A., & Hambrick D. Z. (2010). The relationship between working memory capacity and executive functioning: Evidence for a common executive attention construct. Neuropsychology, 24(2), 222–243. https://doi.org/10.1037/a0017619 20230116 10.1037/a0017619 PMC2852635

[bib64] McCoy S. L., Tun P. A., Cox L. C., Colangelo M., Stewart R. A., & Wingfield A. (2005). Hearing loss and perceptual effort: Downstream effects on older adults' memory for speech. Quarterly Journal of Experimental Psychology Section A: Human Experimental Psychology, 58(1), 22–33. https://doi.org/10.1080/02724980443000151 15881289 10.1080/02724980443000151

[bib65] McLaughlin D. J., Baese-Berk M. M., Bent T., Borrie S. A., & Van Engen K. J. (2018). Coping with adversity: Individual differences in the perception of noisy and accented speech. Attention, Perception, and Psychophysics, 80(6), 1559–1570. https://doi.org/10.3758/s13414-018-1537-4 10.3758/s13414-018-1537-4 29740795

[bib66] Miyake A., Emerson M. J., Padilla F., & Ahn J. C. (2004). Inner speech as a retrieval aid for task goals: The effects of cue type and articulatory suppression in the random task cuing paradigm. Acta Psychologica, 115(2–3), 123–142. https://doi.org/10.1016/j.actpsy.2003.12.004 14962397 10.1016/j.actpsy.2003.12.004

[bib67] Miyake A., & Friedman N. P. (2012). The nature and organization of individual differences in executive functions: Four general conclusions. Current Directions in Psychological Science, 21(1), 8–14. https://doi.org/10.1177/0963721411429458 22773897 10.1177/0963721411429458 PMC3388901

[bib68] Miyake A., Friedman N. P., Emerson M. J., Witzki A. H., Howerter A., & Wager T. D. (2000). The unity and diversity of executive functions and their contributions to complex “frontal lobe” tasks: A latent variable analysis. Cognitive Psychology, 41(1), 49–100. https://doi.org/10.1006/cogp.1999.0734 10945922 10.1006/cogp.1999.0734

[bib69] Moberly A. C., Harris M. S., Boyce L., & Nittrouer S. (2017). Speech recognition in adults with cochlear implants: The effects of working memory, phonological sensitivity, and aging. Journal of Speech, Language, and Hearing Research, 60(4), 1046–1061. https://doi.org/10.1044/2016_JSLHR-H-16-0119 10.1044/2016_JSLHR-H-16-0119 PMC5548076 28384805

[bib70] Moberly A. C., Houston D. M., & Castellanos I. (2016). Non-auditory neurocognitive skills contribute to speech recognition in adults with cochlear implants. Laryngoscope Investigative Otolaryngology, 1(6), 154–162. https://doi.org/10.1002/lio2.38 28660253 10.1002/lio2.38 PMC5467524

[bib71] Moberly A. C., Houston D. M., Harris M. S., Adunka O. F., & Castellanos I. (2017). Verbal working memory and inhibition-concentration in adults with cochlear implants. Laryngoscope Investigative Otolaryngology, 2(5), 254–261. https://doi.org/10.1002/lio2.90 29094068 10.1002/lio2.90 PMC5655567

[bib72] O'Neill E. R., Kreft H. A., & Oxenham A. J. (2019). Cognitive factors contribute to speech perception in cochlear-implant users and age-matched normal-hearing listeners under vocoded conditions. The Journal of the Acoustical Society of America, 146(1), 195–210. https://doi.org/10.1121/1.5116009 31370651 10.1121/1.5116009 PMC6637026

[bib73] Oberauer K. (2002). Access to information in working memory: Exploring the focus of attention. Journal of Experimental Psychology: Learning, Memory, and Cognition, 28(3), 411–421. https://doi.org/10.1037/0278-7393.28.3.411 12018494

[bib74] Pichora-Fuller M. K., Schneider B. A., & Daneman M. (1995). How young and old adults listen to and remember speech in noise. The Journal of the Acoustical Society of America, 97(1), 593–608. https://doi.org/10.1121/1.412282 7860836 10.1121/1.412282

[bib75] Quinlan P. T., Roodenrys S., & Miller L. M. (2017). Serial reconstruction of order and serial recall in verbal short-term memory. Memory and Cognition, 45, 1126–1143. https://doi.org/10.3758/s13421-017-0719-y 28567713 10.3758/s13421-017-0719-y

[bib76] Rey-Mermet A., Gade M., & Oberauer K. (2018). Should we stop thinking about inhibition? Searching for individual and age differences in inhibition ability. Journal of Experimental Psychology: Learning Memory and Cognition, 44(4), 501–526. https://doi.org/10.1037/xlm0000450 10.1037/xlm0000450 28956944

[bib77] Roberts K. L., & Allen H. A. (2016). Perception and cognition in the ageing brain: A brief review of the short- and long-term links between perceptual and cognitive decline. Frontiers in Aging Neuroscience, 8, 39 https://doi.org/10.3389/fnagi.2016.00039 26973514 10.3389/fnagi.2016.00039 PMC4772631

[bib78] Rönnberg J., Lunner T., Ng E. H. N., Lidestam B., Zekveld A. A., Sörqvist P., Lyxell B., Träff U., Yumba W., Classon E., Hällgren M., Larsby B., Signoret C., Pitchora-Fuller M. K., Rudner M., Danielsson H., & Stenfelt S. (2016). Hearing impairment, cognition and speech understanding: Exploratory factor analyses of a comprehensive test battery for a group of hearing aid users, the n200 study. International Journal of Audiology, 55(11), 623–642. https://doi.org/10.1080/14992027.2016.1219775 27589015 10.1080/14992027.2016.1219775 PMC5044772

[bib79] Rönnberg J., Lunner T., Zekveld A., Sörqvist P., Danielsson H., Lyxell B., Dahlström Ö., Signoret C., Stenfelt S., Pichora-Fuller M. K., & Rudner M. (2013). The ease of language understanding (elu) model: Theoretical, empirical, and clinical advances. Frontiers in Systems Neuroscience, 7, 31 https://doi.org/10.3389/fnsys.2013.00031 23874273 10.3389/fnsys.2013.00031 PMC3710434

[bib80] Roodenrys S., Hulme C., Lethbridge A., Hinton M., & Nimmo L. M. (2002). Word-frequency and phonological-neighborhood effects on verbal short-term memory. Journal of Experimental Psychology: Learning, Memory, and Cognition, 28(6), 1019–1034. https://doi.org/10.1037/0278-7393.28.6.1019 10.1037//0278-7393.28.6.1019 12450329

[bib81] Schmiedek F., Oberauer K., Wilhelm O., Süß H. M., & Wittmann W. W. (2007). Individual differences in components of reaction time distributions and their relations to working memory and intelligence. Journal of Experimental Psychology: General, 136(3), 414–429. https://doi.org/10.1037/0096-3445.136.3.414 17696691 10.1037/0096-3445.136.3.414

[bib82] Shelton J. T., Elliott E. M., Matthews R. A., Hill B. D., & Gouvier W. D. (2010). The relationships of working memory, secondary memory, and general fluid intelligence: Working memory is special. Journal of Experimental Psychology: Learning Memory and Cognition, 36(3), 813–820. https://doi.org/10.1037/a0019046 10.1037/a0019046 PMC2864949 20438278

[bib83] Shinn-Cunningham B. G., & Best V. (2008). Selective attention in normal and impaired hearing. Trends in Amplification, 12(4), 283–299. https://doi.org/10.1177/1084713808325306 18974202 10.1177/1084713808325306 PMC2700845

[bib84] Shipstead Z., Harrison T. L., & Engle R. W. (2016). Working memory capacity and fluid intelligence. Perspectives on Psychological Science, 11(6), 771–799. https://doi.org/10.1177/1745691616650647 27899724 10.1177/1745691616650647

[bib85] Shipstead Z., Lindsey D. R. B., Marshall R. L., & Engle R. W. (2014). The mechanisms of working memory capacity: Primary memory, secondary memory, and attention control. Journal of Memory and Language, 72, 116–141. https://doi.org/10.1016/j.jml.2014.01.004

[bib86] Smith G. N., Pisoni D. B., & Kronenberger W. G. (2019). High-variability sentence recognition in long-term cochlear implant users: Associations with rapid phonological coding and executive functioning. Ear and Hearing, 40(5), 1149–1161. https://doi.org/10.1097/AUD.0000000000000691 30601227 10.1097/AUD.0000000000000691 PMC7504907

[bib87] Smith S. L., Pichora-Fuller M. K., & Alexander G. (2016). Development of the word auditory recognition and recall measure. Ear and Hearing, 37(6), e360–e376. https://doi.org/10.1097/AUD.0000000000000329 27438869 10.1097/AUD.0000000000000329

[bib88] Storkel H. L. (2013). A corpus of consonant–vowel–consonant (CVC) real words and nonwords: Comparison of phonotactic probability, neighborhood density, and consonant age-of-acquisition. Behavioral Research Methods, 45(4), 1159–1167. https://doi.org/10.3758/s13428-012-0309-7 10.3758/s13428-012-0309-7 PMC3633677 23307574

[bib89] Stroop J. R. (1935). Studies of interference in serial verbal reactions. Journal of Experimental Psychology, 18(6), 643–662. https://doi.org/10.1037/h0054651

[bib90] Tamati T. N., Gilbert J. L., & Pisoni D. B. (2013). Some factors underlying individual differences in speech recognition on PRESTO: A first report. Journal of the American Academy of Audiology, 24(7), 616–634. https://doi.org/10.3766/jaaa.24.7.10 24047949 10.3766/jaaa.24.7.10 PMC3799882

[bib91] Tulsky D. S., & Price L. R. (2003). The joint WAIS-III and WMS-III factor structure: Development and cross-validation of a six-factor model of cognitive functioning. Psychological Assessment, 15(2), 149–162. https://doi.org/10.1037/1040-3590.15.2.149 12847775 10.1037/1040-3590.15.2.149

[bib92] Unsworth N., & Engle R. W. (2006). Simple and complex memory spans and their relation to fluid abilities: Evidence from list-length effects. Journal of Memory and Language, 54(1), 68–80. https://doi.org/10.1016/j.jml.2005.06.003

[bib93] Unsworth N., & Engle R. W. (2007a). On the division of short-term and working memory: An examination of simple and complex span and their relation to higher order abilities. Psychological Bulletin, 133(6), 1038–1066. https://doi.org/10.1037/0033-2909.133.6.1038 17967093 10.1037/0033-2909.133.6.1038

[bib94] Unsworth N., & Engle R. W. (2007b). The nature of individual differences in working memory capacity: Active maintenance in primary memory and controlled search from secondary memory. Psychological Review, 114(1), 104–132. https://doi.org/10.1037/0033-295X.114.1.104 17227183 10.1037/0033-295X.114.1.104

[bib95] Unsworth N., Spillers G. J., & Brewer G. A. (2009). Examining the relations among working memory capacity, attention control, and fluid intelligence from a dual-component framework. Psychology Science Quarterly, 51(4), 388–402.

[bib96] van Rooij J. C. G. M., & Plomp R. (1990). Auditive and cognitive factors in speech perception by elderly listeners. II: Multivariate analyses. The Journal of the Acoustical Society of America, 88(6), 2611–2624. https://doi.org/10.1121/1.399981 2283434 10.1121/1.399981

[bib97] Veneman C. E., Gordon-Salant S., Matthews L. J., & Dubno J. R. (2013). Age and measurement time of day effects on speech recognition in noise. Ear and Hearing, 34(3), 288–299. https://doi.org/10.1097/AUD.0b013e31826d0b81 23187606 10.1097/AUD.0b013e31826d0b81 PMC3587027

[bib98] Wechsler D. (2011). WASI-II: Wechsler Abbreviated Scale of Intelligence–Second Edition. The Psychological Corporation https://doi.org/10.1037/t15171-000

[bib99] White C. N., Curl R. A., & Sloane J. F. (2016). Using decision models to enhance investigations of individual differences in cognitive neuroscience. Frontiers in Psychology, 7, 81 https://doi.org/10.3389/fpsyg.2016.00081 26903896 10.3389/fpsyg.2016.00081 PMC4746304

[bib100] White C. N., Servant M., & Logan G. D. (2018). Testing the validity of conflict drift-diffusion models for use in estimating cognitive processes: A parameter-recovery study. Psychonomic Bulletin and Review, 25(1), 286–301. https://doi.org/10.3758/s13423-017-1271-2 28357629 10.3758/s13423-017-1271-2 PMC5788738

[bib101] Wilhelm O., Hildebrandt A., & Oberauer K. (2013). What is working memory capacity, and how can we measure it? Frontiers in Psychology, 4, 433 https://doi.org/10.3389/fpsyg.2013.00433 23898309 10.3389/fpsyg.2013.00433 PMC3721021

[bib102] Wingfield A., Amichetti N. M., & Lash A. (2015). Cognitive aging and hearing acuity: Modeling spoken language comprehension. Frontiers in Psychology, 6, 684 https://doi.org/10.3389/fpsyg.2015.00684 26124724 10.3389/fpsyg.2015.00684 PMC4462993

[bib103] Woods D. L., Kishiyama M. M., Yund E. W., Herron T. J., Edwards B., Hink R. F., & Reed B. (2011). Improving digit span assessment of short-term verbal memory. Journal of Clinical Experimental Neuropsychology, 33(1), 101–111. https://doi.org/10.1080/13803395.2010.493149 20680884 10.1080/13803395.2010.493149 PMC2978794

[bib104] Xia J., Kalluri S., Micheyl C., & Hafter E. (2017). Continued search for better prediction of aided speech understanding in multi-talker environments. The Journal of the Acoustical Society of America, 142(4), 2386–2399. https://doi.org/10.1121/1.5008498 29092591 10.1121/1.5008498

[bib105] Yoho S. E., Healy E. W., Youngdahl C. L., Barrett T. S., & Apoux F. (2018). Speech-material and talker effects in speech band importance. The Journal of the Acoustical Society of America, 143(3), 1417–1426. https://doi.org/10.1121/1.5026787 29604719 10.1121/1.5026787 PMC5851785

