# Supplementary material for: Serial Recall Predicts Vocoded Sentence Recognition Across Spectral Resolutions
Source: J Speech Lang Hear Res. 2020 Mar 26;63(4):1282–98. doi: 10.1044/2020_JSLHR-19-00319 (PMC7242981; doi:10.1044/2020_JSLHR-19-00319)
Supplement: Supplemental Material S1 [file JSLHR-63-1282-s001.zip › Supplemental Material/EF Tasks/colorshapetask/exgauss estimation/demo/html/fminsearchbnd_demo.html]

fminsearchbnd\_demo


 


## Contents

- Optimization of a simple (Rosenbrock) function, with no constraints
- Only lower bound constraints
- Only upper bound constraints
- Dual constraints
- Mixed constraints
- Provide your own fminsearch options
- Exactly fix one variable, constrain some others, and set a tolerance
- All the standard outputs from fminsearch are still returned

## Optimization of a simple (Rosenbrock) function, with no constraints

```
rosen = @(x) (1-x(1)).^2 + 105*(x(2)-x(1).^2).^2;

% With no constraints, operation simply passes through
% directly to fminsearch. The solution should be [1 1]
xsol = fminsearchbnd(rosen,[3 3])
```

```
xsol =

      0.99998      0.99995
```

## Only lower bound constraints

```
xsol = fminsearchbnd(rosen,[3 3],[2 2])
```

```
xsol =

            2            4
```

## Only upper bound constraints

```
xsol = fminsearchbnd(rosen,[-5 -5],[],[0 0])
```

```
xsol =

  -1.0447e-13  -1.4451e-08
```

## Dual constraints

```
xsol = fminsearchbnd(rosen,[2.5 2.5],[2 2],[3 3])
```

```
xsol =

            2            3
```

## Mixed constraints

```
xsol = fminsearchbnd(rosen,[0 0],[2 -inf],[inf 3])
```

```
xsol =

            2            3
```

## Provide your own fminsearch options

```
opts = optimset('fminsearch');
opts.Display = 'iter';
opts.TolX = 1.e-12;
opts.MaxFunEvals = 100;

n = [10,5];
H = randn(n);
H=H'*H;
Quadraticfun = @(x) x*H*x';

% Global minimizer is at [0 0 0 0 0].
% Set all lower bound constraints, all of which will
% be active in this test.
LB = [.5 .5 .5 .5 .5];
xsol = fminsearchbnd(Quadraticfun,[1 2 3 4 5],LB,[],opts)
```

```
 Iteration   Func-count     min f(x)         Procedure
     0            1          173.731         
     1            6          172.028         initial simplex
     2            8          162.698         expand
     3            9          162.698         reflect
     4           11          151.902         expand
     5           13          138.235         expand
     6           14          138.235         reflect
     7           16          126.604         expand
     8           17          126.604         reflect
     9           19          97.3266         expand
    10           20          97.3266         reflect
    11           21          97.3266         reflect
    12           22          97.3266         reflect
    13           24          73.7178         expand
    14           25          73.7178         reflect
    15           26          73.7178         reflect
    16           28          50.8236         expand
    17           29          50.8236         reflect
    18           31          41.6294         expand
    19           33          30.4252         expand
    20           34          30.4252         reflect
    21           36           27.782         reflect
    22           37           27.782         reflect
    23           39           27.782         contract inside
    24           41          22.6509         reflect
    25           42          22.6509         reflect
    26           43          22.6509         reflect
    27           44          22.6509         reflect
    28           45          22.6509         reflect
    29           47          21.0211         reflect
    30           48          21.0211         reflect
    31           49          21.0211         reflect
    32           51          21.0211         contract inside
    33           52          21.0211         reflect
    34           54          20.7613         contract inside
    35           55          20.7613         reflect
    36           56          20.7613         reflect
    37           57          20.7613         reflect
    38           59          20.6012         contract inside
    39           61          20.5324         contract inside
    40           63          20.4961         contract inside
    41           65          20.3886         contract inside
    42           67          20.2121         reflect
    43           69          20.0876         contract inside
    44           71          19.9164         reflect
    45           72          19.9164         reflect
    46           74          19.9164         contract inside
    47           76          19.9164         contract outside
    48           78          19.3349         expand
    49           80          19.3349         contract inside
    50           81          19.3349         reflect
    51           82          19.3349         reflect
    52           84          18.8721         expand
    53           85          18.8721         reflect
    54           87          18.6427         expand
    55           89          17.4548         expand
    56           90          17.4548         reflect
    57           92          16.0113         expand
    58           93          16.0113         reflect
    59           94          16.0113         reflect
    60           96          14.6134         expand
    61           98          12.5445         expand
    62           99          12.5445         reflect
    63          101          10.7311         expand
 
Exiting: Maximum number of function evaluations has been exceeded
         - increase MaxFunEvals option.
         Current function value: 10.731146 


xsol =

       1.7022       1.0787       1.2034       0.5006      0.64666
```

## Exactly fix one variable, constrain some others, and set a tolerance

```
opts = optimset('fminsearch');
opts.TolFun = 1.e-12;

LB = [-inf 2 1 -10];
UB = [ inf  inf 1  inf];
xsol = fminsearchbnd(@(x) norm(x),[1 3 1 1],LB,UB,opts)
```

```
xsol =

  -4.9034e-07            2            1   5.1394e-07
```

## All the standard outputs from fminsearch are still returned

```
[xsol,fval,exitflag,output] = fminsearchbnd(@(x) norm(x),[1 3 1 1],LB,UB)
```

```
xsol =

   3.1094e-05            2            1  -5.1706e-05


fval =

       2.2361


exitflag =

     1


output = 

    iterations: 77
     funcCount: 138
     algorithm: 'Nelder-Mead simplex direct search'
       message: [1x194 char]
```

Published with MATLAB® 7.0.1
