# Supplementary material for: Serial Recall Predicts Vocoded Sentence Recognition Across Spectral Resolutions
Source: J Speech Lang Hear Res. 2020 Mar 26;63(4):1282–98. doi: 10.1044/2020_JSLHR-19-00319 (PMC7242981; doi:10.1044/2020_JSLHR-19-00319)
Supplement: Supplemental Material S1 [file JSLHR-63-1282-s001.zip › Supplemental Material/EF Tasks/colorshapetask/exgauss estimation/demo/html/fminsearchcon_demo.html]

fminsearchcon\_demo


 


## Contents

- Optimization of a simple (Rosenbrock) function
- Only lower bound constraints
- Only upper bound constraints
- Dual constraints
- Mixed constraints
- Fix a variable as constant, x(2) == 3
- Linear inequality, x(1) + x(2) <= 1
- Nonlinear inequality, norm(x) <= 1
- Minimize a linear objective, subject to a nonlinear constraints.
- Provide your own fminsearch options
- Exactly fix one variable, constrain some others, and set a tolerance
- All the standard outputs from fminsearch are still returned

## Optimization of a simple (Rosenbrock) function

```
rosen = @(x) (1-x(1)).^2 + 105*(x(2)-x(1).^2).^2;

% With no constraints, operation simply passes through
% directly to fminsearch. The solution should be [1 1]
xsol = fminsearchcon(rosen,[3 3])
```

```
xsol =

      0.99998      0.99995
```

## Only lower bound constraints

```
xsol = fminsearchcon(rosen,[3 3],[2 2])
```

```
xsol =

            2            4
```

## Only upper bound constraints

```
xsol = fminsearchcon(rosen,[-5 -5],[],[0 0])
```

```
xsol =

  -1.0447e-13  -1.4451e-08
```

## Dual constraints

```
xsol = fminsearchcon(rosen,[2.5 2.5],[2 2],[3 3])
```

```
xsol =

            2            3
```

## Mixed constraints

```
xsol = fminsearchcon(rosen,[0 0],[2 -inf],[inf 3])
```

```
xsol =

            2            3
```

## Fix a variable as constant, x(2) == 3

```
fminsearchcon(rosen,[3 3],[-inf 3],[inf,3])
```

```
ans =

       1.7314            3
```

## Linear inequality, x(1) + x(2) <= 1

```
fminsearchcon(rosen,[0 0],[],[],[1 1],1)
```

```
ans =

       0.6187       0.3813
```

## Nonlinear inequality, norm(x) <= 1

```
fminsearchcon(rosen,[0 0],[],[],[],[],@(x) norm(x) - 1)
```

```
ans =

      0.78633      0.61778
```

## Minimize a linear objective, subject to a nonlinear constraints.

```
fun = @(x) x*[-2;1];
nonlcon = @(x) [norm(x) - 1;sin(sum(x))];
fminsearchcon(fun,[0 0],[],[],[],[],nonlcon)
```

```
ans =

      0.70707     -0.70713
```

## Provide your own fminsearch options

```
opts = optimset('fminsearch');
opts.Display = 'iter';
opts.TolX = 1.e-12;
opts.MaxFunEvals = 100;

n = [10,5];
H = randn(n);
H=H'*H;
Quadraticfun = @(x) x*H*x';

% Global minimizer is at [0 0 0 0 0].
% Set all lower bound constraints, all of which will
% be active in this test.
LB = [.5 .5 .5 .5 .5];
xsol = fminsearchcon(Quadraticfun,[1 2 3 4 5],LB,[],[],[],[],opts)
```

```
 Iteration   Func-count     min f(x)         Procedure
     0            1          351.442         
     1            6          351.442         initial simplex
     2            8          296.007         expand
     3            9          296.007         reflect
     4           10          296.007         reflect
     5           11          296.007         reflect
     6           13          248.853         expand
     7           15          209.119         expand
     8           16          209.119         reflect
     9           17          209.119         reflect
    10           19          171.673         expand
    11           21          132.166         expand
    12           22          132.166         reflect
    13           23          132.166         reflect
    14           24          132.166         reflect
    15           25          132.166         reflect
    16           27          129.206         reflect
    17           28          129.206         reflect
    18           30          124.943         reflect
    19           32          124.943         contract inside
    20           34          123.809         reflect
    21           36          107.233         expand
    22           38          107.233         contract outside
    23           39          107.233         reflect
    24           41          98.5266         expand
    25           42          98.5266         reflect
    26           44          94.0621         expand
    27           46          87.7403         expand
    28           48          86.0587         reflect
    29           49          86.0587         reflect
    30           50          86.0587         reflect
    31           51          86.0587         reflect
    32           53          85.8138         reflect
    33           55          85.8138         contract inside
    34           57          73.0352         expand
    35           59          73.0352         contract inside
    36           60          73.0352         reflect
    37           61          73.0352         reflect
    38           62          73.0352         reflect
    39           64          64.9347         expand
    40           66          62.3724         expand
    41           68          62.3126         reflect
    42           69          62.3126         reflect
    43           71          57.2349         reflect
    44           73           49.999         expand
    45           74           49.999         reflect
    46           75           49.999         reflect
    47           76           49.999         reflect
    48           78          48.1326         reflect
    49           79          48.1326         reflect
    50           81          48.1326         contract inside
    51           83          46.1196         reflect
    52           85          46.1196         contract inside
    53           87          43.1862         reflect
    54           88          43.1862         reflect
    55           90          43.1862         contract inside
    56           91          43.1862         reflect
    57           92          43.1862         reflect
    58           94          42.6876         reflect
    59           95          42.6876         reflect
    60           97          42.6876         contract outside
    61           99          41.6298         reflect
    62          100          41.6298         reflect
 
Exiting: Maximum number of function evaluations has been exceeded
         - increase MaxFunEvals option.
         Current function value: 41.629797 


xsol =

        1.652      0.54831       2.4744       1.2291      0.56971
```

## Exactly fix one variable, constrain some others, and set a tolerance

```
opts = optimset('fminsearch');
opts.TolFun = 1.e-12;

LB = [-inf 2 1 -10];
UB = [ inf  inf 1  inf];
xsol = fminsearchcon(@(x) norm(x),[1 3 1 1],LB,UB,[],[],[],opts)
```

```
xsol =

  -4.9034e-07            2            1   5.1394e-07
```

## All the standard outputs from fminsearch are still returned

```
[xsol,fval,exitflag,output] = fminsearchcon(@(x) norm(x),[1 3 1 1],LB,UB)
```

```
xsol =

   3.1094e-05            2            1  -5.1706e-05


fval =

       2.2361


exitflag =

     1


output = 

    iterations: 77
     funcCount: 138
     algorithm: 'Nelder-Mead simplex direct search'
       message: [1x194 char]
```

Published with MATLAB® 7.0.1
