# Supplementary material for: Serial Recall Predicts Vocoded Sentence Recognition Across Spectral Resolutions
Source: J Speech Lang Hear Res. 2020 Mar 26;63(4):1282–98. doi: 10.1044/2020_JSLHR-19-00319 (PMC7242981; doi:10.1044/2020_JSLHR-19-00319)
Supplement: Supplemental Material S1 [file JSLHR-63-1282-s001.zip › Supplemental Material/EF Tasks/colorshapetask/exgauss estimation/doc/Understanding_fminsearchbnd.rtf]

Understanding fminsearchbndJohn D'Erricowoodchips@rochester.rr.comFminsearchbnd is really quite simple in concept. I've implemented lower and upper bound constraints by the careful use of transformations of the variables. In turn, the optimization engine is itself fminsearch itself.Since fminsearch does not allow bound constraints, the trick is to insert a wrapper function around the user supplied objective function. I've done all the work, so all you, as the user, needs to do is supply a set of bounds. All the other arguments are identical to that which fminsearch would have expected. There are several classes of bound constraints one might consider. Simple lower bound constraints:	LB(i) <= x(i)Upper bound constraints:	x(i) <= UB(i)Dual constraints	LB(i) <= x(i) <= UB(i)Two other classes that I allow are fully unbounded variables, and a dual constraint where the lower and upper bounds are identical. In essence, this last class of constraint fixes the variable at the given level. Of course, internally in fminsearchbnd, I just completely remove that variable from the optimization, so fminsearch never sees the variable at all.The bounded variables are transformed such that fminsearch itself sees a fully unconstrained problem. For example, in the case of a variable bounded on the lower end by LB(i), I use the transformation	x(i) = LB(i) + z(i)^2The variable z(i) is fully unconstrained, but since the square of z(i) is always non-negative (for real z), then x(i) must necessarily be always greater than or equal to LB(i). Likewise, a pure upper bound constraint is implemented as	x(i) = UB(i) - z(i)^2Clearly, x(i) in this case can never rise above UB(i). And finally, the dual bounded variable is handled by a trigonometric transformation,	x(i) = LB(i) + (UB(i) - LB(i))*(sin(z(i))+1)/2In this last case, I do absolutely enforce the requirement that LB(i) <= x(i) <= UB(i), since the vagaries of floating point arithmetic might sometimes cause those bounds to be subtly exceeded.Multiple solutions due to the transformationsAn artifact of the transformations used is the creation of multiple solutions to a problem that at one time may well have had a unique solution. While the presence of multiple local solutions is often a problem for an optimizer, each of these introduced solutions are fully equivalent. It matters not in the least which one is found.Alternative choices for the transformationsI have occasionally seen it suggested that one use a sin(z)^2 transformation instead of the chosen form based on sin(z). My own feeling is that either could be used to roughly equal advantage, but that the sin^2 transformation may be slightly more nonlinear, causing subtly more problems in terms of floating point arithmetic. Similarly, I've tried other one sided and two sided transformations. A two sided transformation that I found to be of interest utilized atan(z). My testing showed that it was often more slowly convergent near the bounds, taking more iterations to converge. The atan transformation was a good choice as a way to implement exclusive bounds (see below.)Inclusive versus exclusive boundsA feature of the bound constraints that I have chosen to implement in fminsearchbnd is that they are inclusive bounds. That is, these constraints allow the boundary value itself to be achieved. Exclusive constraints would correspond to the strict inequalities, < and >. Why is this difference between inclusive and exclusive bound constraints an issue? As I said, fminsearchbnd allows its bounds to be fully achieved. So if your objective function includes an evaluation of log(x), where x is constrained to be greater than or equal to zero, then Matlab will generate a singularity.>> log(0)Warning: Log of zero.ans =  -InfAn exclusive bound at zero would have prevented such an error. I've chosen not to implement them in that form however, as the necessary transformations tend to be somewhat more intractable, converging with less rapidity in practice. Also, the interface would have been more complex had I allowed the user to specify the actual boundary type for each constraint.All of this means that if you really need exclusive bounds, then you must offset your bound limits by a small amount.Starting values, infeasible starting values, tolerances, etc.The transformations chosen for fminsearchbnd are all simply invertible. This allows the user supplied starting values for each variable (prior to any transformation) to be simply mapped back to a corresponding value. Infeasible starting values are simply resolved for the bound constrained problem by simply clipping to the bounded domain.A more difficult issue is the question of tolerances on the parameters, that is, TolX. The nonlinear transformations mean that fminsearch itself will see only the transformed parameters, not the parameters in their real domain. As I've implemented fminsearchbnd as an overlay to fminsearch itself, there is no simple way to provide explicit control over the variable tolerances without re-writing fminsearch. Limitations of fminsearchbndWhat does fminsearchbnd NOT do? You cannot provide general linear/nonlinear equality or inequality constraints, as are provided by fmincon, or lsqlin. Only simple bound constraints are allowed.
