# Supplementary material for: Serial Recall Predicts Vocoded Sentence Recognition Across Spectral Resolutions
Source: J Speech Lang Hear Res. 2020 Mar 26;63(4):1282–98. doi: 10.1044/2020_JSLHR-19-00319 (PMC7242981; doi:10.1044/2020_JSLHR-19-00319)
Supplement: Supplemental Material S1 [file JSLHR-63-1282-s001.zip › Supplemental Material/EF Tasks/colorshapetask/exgauss estimation/doc/Understanding_fminsearchcon.rtf]

Understanding fminsearchconJohn D'Erricowoodchips@rochester.rr.comFminsearchcon is a simple extension of fminsearchbnd, which implemented simple bound constraints on top of fminsearch. (See Understanding_fminsearchbnd.rtf for an explanation of bound constraints.) Fminsearchcon goes one step further, allowing general linear inequality constraints and nonlinear inequality constraints. These constraints are implemented as penalty functions, thus if fminsearchbnd would have tried to evaluate your objective function outside the bounds, then I intercept the call BEFORE I allow it to evaluate the objective function, I then return inf to fminsearch. The expectation is that your objective function will never be evaluated outside of the supplied constraints.Limitations of fminsearchconWhat does fminsearchcon NOT do? The only class of constraint that I have not implemented are general linear or nonlinear EQUALITY constraints. These coonstraints are incompatible with the bound transformations used. As well, fminsearch would not in general work well when confined to a nonlinear manifold.Do NOT attempt to implement a nonlinear equality constraint using a pair of matched inequalities. Thus    c(x) <= 0   -c(x) <= 0You will not be happy with the results in general.
