# Supplementary material for: Serial Recall Predicts Vocoded Sentence Recognition Across Spectral Resolutions
Source: J Speech Lang Hear Res. 2020 Mar 26;63(4):1282–98. doi: 10.1044/2020_JSLHR-19-00319 (PMC7242981; doi:10.1044/2020_JSLHR-19-00319)
Supplement: Supplemental Material S1 [file JSLHR-63-1282-s001.zip › Supplemental Material/EF Tasks/colorshapetask/sc_blockedintro2.htm]

COLOR SHAPE TASK instructions


Let�s try a few practice rounds.

You will be provided feedback if your response is incorrect.

  
  

Press SPACEBAR to continue
