# Supplementary material for: Serial Recall Predicts Vocoded Sentence Recognition Across Spectral Resolutions
Source: J Speech Lang Hear Res. 2020 Mar 26;63(4):1282–98. doi: 10.1044/2020_JSLHR-19-00319 (PMC7242981; doi:10.1044/2020_JSLHR-19-00319)
Supplement: Supplemental Material S1 [file JSLHR-63-1282-s001.zip › Supplemental Material/EF Tasks/colorshapetask/sc_blocked_bivalentcolortest.htm]

COLOR SHAPE TASK instructions


# TASK: Identify the COLOR

RED: <%values.responsekey\_red\_label%> - <%expressions.buttoninstruct1%>

GREEN: <%values.responsekey\_green\_label%> - <%expressions.buttoninstruct1%>

  
  

Please try to respond as quickly and as accurately as possible.

  
  

Press SPACEBAR to start.
