# Supplementary material for: Serial Recall Predicts Vocoded Sentence Recognition Across Spectral Resolutions
Source: J Speech Lang Hear Res. 2020 Mar 26;63(4):1282–98. doi: 10.1044/2020_JSLHR-19-00319 (PMC7242981; doi:10.1044/2020_JSLHR-19-00319)
Supplement: Supplemental Material S1 [file JSLHR-63-1282-s001.zip › Supplemental Material/EF Tasks/colorshapetask/sc_blocked_colorshapetraining.htm]

COLOR SHAPE TASK instructions


For this practice round, you are presented shapes without color OR simple squares of color.

  

TASK:  
=> When you see a shape, identify the shape.  
=> When you see a colored square, identify the color.

red / *<%expressions.redshapemapping%>*: <%values.responsekey\_red\_label%> - <%expressions.buttoninstruct1%>

green / *<%expressions.greenshapemapping%>*: <%values.responsekey\_green\_label%> - <%expressions.buttoninstruct1%>

  

Please try to respond as quickly and as accurately as possible.

  
  

Press SPACEBAR to start.
