# Supplementary material for: Serial Recall Predicts Vocoded Sentence Recognition Across Spectral Resolutions
Source: J Speech Lang Hear Res. 2020 Mar 26;63(4):1282–98. doi: 10.1044/2020_JSLHR-19-00319 (PMC7242981; doi:10.1044/2020_JSLHR-19-00319)
Supplement: Supplemental Material S1 [file JSLHR-63-1282-s001.zip › Supplemental Material/EF Tasks/colorshapetask/sc_blocked_testintro.htm]

COLOR SHAPE TASK instructions


Practice is over and the actual SHAPES and COLORS game is about to start:

  

You will be presented *CIRCLES* and *TRIANGLES* that are superimposed on red
and green color patches.

  

The task instructions will tell you if you need to identify the COLOR or the *SHAPE* of the presented objects.

There is **not** going to be any feedback anymore if you make a mistake.

  
  

Press SPACEBAR to continue.
