# Supplementary material for: Serial Recall Predicts Vocoded Sentence Recognition Across Spectral Resolutions
Source: J Speech Lang Hear Res. 2020 Mar 26;63(4):1282–98. doi: 10.1044/2020_JSLHR-19-00319 (PMC7242981; doi:10.1044/2020_JSLHR-19-00319)
Supplement: Supplemental Material S1 [file JSLHR-63-1282-s001.zip › Supplemental Material/EF Tasks/colorshapetask/sc_generalintro.htm]

COLOR SHAPE TASK instructions


You are going to play a game called SHAPES AND COLORS.

  
  

In the game you will see objects. These objects can be *CIRCLES* or *TRIANGLES* that are superimposed on green
or red squares.

  

Sometimes you will need to indicate whether the COLOR is GREEN or RED.

Other times you will need to indicate whether the SHAPE is a ◯ *CIRCLE* or a △ *TRIANGLE*.

  
  
  

Press SPACEBAR to continue
