# Supplementary material for: Serial Recall Predicts Vocoded Sentence Recognition Across Spectral Resolutions
Source: J Speech Lang Hear Res. 2020 Mar 26;63(4):1282–98. doi: 10.1044/2020_JSLHR-19-00319 (PMC7242981; doi:10.1044/2020_JSLHR-19-00319)
Supplement: Supplemental Material S1 [file JSLHR-63-1282-s001.zip › Supplemental Material/EF Tasks/colorshapetask/sc_mixedintro1.htm]

COLOR SHAPE TASK instructions


The COLOR and SHAPE Game will change slightly for the next rounds.

  

In the past rounds you were told before each round whether to identify the color or the shape of the presented objects.

For the next rounds you will **not** be told before each round whether to identify the color or the shape of the presented objects.
Instead you will briefly see either the words 'COLOR' or 'SHAPE' or the letters 'C' (for Color) or 'S' (for Shape) on the screen **before each object**
is presented.

  

If you see 'COLOR' or 'C', identify whether the object is RED or GREEN.

If you see 'SHAPE' or 'S', identify whether the object is a ◯ *CIRCLE* or a △ *TRIANGLE*.

  

Please try to respond as quickly and as accurately as possible.

  
  

Press SPACEBAR to continue.
