# Supplementary material for: Serial Recall Predicts Vocoded Sentence Recognition Across Spectral Resolutions
Source: J Speech Lang Hear Res. 2020 Mar 26;63(4):1282–98. doi: 10.1044/2020_JSLHR-19-00319 (PMC7242981; doi:10.1044/2020_JSLHR-19-00319)
Supplement: Supplemental Material S1 [file JSLHR-63-1282-s001.zip › Supplemental Material/EF Tasks/colorshapetask/sc_mixed_wordtest.htm]

COLOR SHAPE TASK instructions


For this test round, either the word SHAPE or the word COLOR will appear briefly before each object.

When you see the word SHAPE, identify whether the object is a a ◯ *CIRCLE* or a △ *TRIANGLE*.

When you see the word COLOR, identify whether the object is red or green.

red / *<%expressions.redshapemapping%>*: <%values.responsekey\_red\_label%> - <%expressions.buttoninstruct1%>

green / *<%expressions.greenshapemapping%>*: <%values.responsekey\_green\_label%> - <%expressions.buttoninstruct1%>

  

Please try to respond as quickly and as accurately as possible.

  
  

Press SPACEBAR to start.
