# Supplementary material for: Serial Recall Predicts Vocoded Sentence Recognition Across Spectral Resolutions
Source: J Speech Lang Hear Res. 2020 Mar 26;63(4):1282–98. doi: 10.1044/2020_JSLHR-19-00319 (PMC7242981; doi:10.1044/2020_JSLHR-19-00319)
Supplement: Supplemental Material S1 [file JSLHR-63-1282-s001.zip › Supplemental Material/EF Tasks/stroopwithvoicerecording/stroopinstructions1.htm]

Stroop Instructions


In the following trials you will see words presented in different colors. Your task is to indicate the COLOR in which each word is printed in
while ignoring what the words actually say.

Indicate the color of the word by pressing either of the following <%expressions.buttoninstruct1%>:

- - d for **red** words
- - f for **green** words
- - j for **blue** words
- - k for **black** words

  

Example: if you see the word **RED** printed in the color GREEN press 'f' for green words regardless of the meaning of the word.

Try to respond as quickly and accurately as you can, because you will be timed. If an incorrect response is made, a red X will be flashed onto the screen.
